# Critical Roles of *O*‐Acetylation Revealed by Synthetic Trisaccharide Antigens for a Broad‐Spectrum Anti‐*Salmonella* Vaccine

**DOI:** 10.1002/advs.77776

**Published:** 2026-09-16

**Authors:** Xingling Pan, Soham Maity, Herbert Kavunja, Yuvarani Murali, Ting‐An Chen, Chang‐xin Huo, Scott M. Baliban, Xuefei Huang

**Affiliations:** ^1^ Department of Chemistry Michigan State University East Lansing Michigan USA; ^2^ Institute For Quantitative Health Science & Engineering Michigan State University East Lansing Michigan USA; ^3^ Iaso Therapeutics Inc. East Lansing Michigan USA; ^4^ Center For Vaccine Development and Global Health University of Maryland School of Medicine Baltimore USA; ^5^ Department of Biomedical Engineering Michigan State University East Lansing Michigan USA

**Keywords:** broad‐spectrum, O‐acetylation, O‐polysaccharide, Salmonella, synthesis, vaccines

## Abstract

*Salmonella* infections are a major global health burden, exacerbated by rampant antimicrobial resistance and the narrow serovar coverage of current vaccines. To overcome the limitations of current vaccine design, structurally defined trisaccharides derived from *Salmonella* O‐polysaccharide backbones composed of D‐mannose‐α(1→4)‐L‐rhamnose‐α(1→3)‐D‐galactose‐α(1→2) sequence and differing only in *O*‐acetylation patterns on the rhamnose were synthesized and conjugated with mQβ virus‐like particles. The resulting glycoconjugates elicited robust glycan‐specific antibody responses. Interestingly, while the vaccine based on the non‐acetylated antigen was not protective against typhoidal strains of *Salmonella*, rabbit antisera induced by the diacetylated rhamnose containing trisaccharide conjugate provided effective protection to mice against lethal challenges by four major pathogenic *Salmonella* serovars including both typhoidal and non‐typhoidal serovars. Mechanistic studies revealed that antigen di‐*O*‐acetylation significantly enhanced complement deposition and opsonophagocytosis of bacteria by the antibodies produced. This is the first time that a vaccine based on a single antigen protected against all four major pathogenic serovars to humans, opening new ground for broad spectrum anti‐*Salmonella* vaccine design. In addition, this suggests introduction of defined acetylation to synthetic *O*‐antigen can be a promising strategy for vaccine development as many bacterial O‐polysaccharides are modified by acetates in nature.

## Graphical abstract

A synthetic glycoconjugate vaccine based on a diacetylated *Salmonella* trisaccharide elicits broadly protective antibodies against four major human pathogenic serovars. Mechanistic studies identify 2,3‐di‐*O*‐acetylation of rhamnose as a critical determinant of antigen recognition and breadth of protection, establishing a new strategy for broad‐spectrum anti‐*Salmonella* vaccine design.

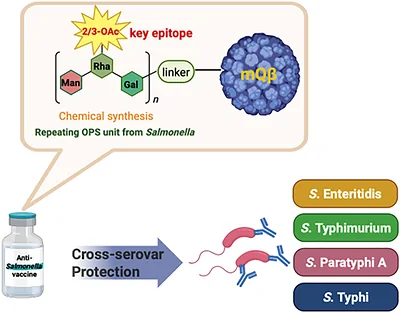

## 1 Introduction

*Salmonella enterica* subspecies *enterica*, a major foodborne and zoonotic pathogen within the family Enterobacteriaceae, is a leading cause of infectious diarrhea worldwide [1]. The *Salmonella enterica* serotypes are broadly classified into typhoidal and nontyphoidal *Salmonella* (NTS). The human‐restricted typhoidal serovars, *Salmonella* Typhi (*S*. Typhi) and *Salmonella* Paratyphi (*S*. Paratyphi), cause enteric fever, with an estimated 9.3 million cases and 107 500 deaths globally in 2021 [2]. In comparison, NTS infections are far more widespread, causing 93.8 million illnesses annually including 80.3 million foodborne cases [3], with invasive NTS (iNTS) disease accounting for 3.4 million cases and 681,316 deaths each year [4]. Among NTS serovars, *Salmonella* Typhimurium (*S*. Typhimurium) and *Salmonella* Enteritidis (*S*. Enteritidis) are the most prevalent [5, 6]. NTS is a leading cause of foodborne mortality in the United States [7] and is the second most common zoonotic infection in Europe [8].

*Salmonella* infections are typically treated with antibiotics. The tremendous public health burden caused by salmonellosis is exacerbated by the alarming prevalence of multidrug‐resistant (MDR) *Salmonella* strains. Resistance has been observed even for antibiotics reserved for severe infections‐including those for invasive cases and infections in immunocompromised individuals, complicating treatment and raising serious public health concerns [9]. As a result, the World Health Organization has listed *Salmonella* as high priority on their bacterial priority pathogen lists. These challenges underscore the urgent need for preventive strategies, especially the development of effective, affordable, and broad‐spectrum anti‐*Salmonella* vaccines [10].

Despite decades of research, the development of a *Salmonella* vaccine capable of protecting against multiple pathogenic serovars remains a formidable challenge. Currently licensed typhoid vaccines, including the live attenuated Ty21a vaccine and the parenteral Vi capsular polysaccharide (CPS) based formulations [11, 12, 13, 14, 15], are restricted to a narrow subset of serovars, leaving large populations susceptible to *Salmonella* infection. For example, Vi CPS vaccines are not effective against Vi‐negative strains, including Vi‐negative *S*. Typhi, or other major pathogenic serovars such as *S*. Paratyphi A, *S*. Enteritidis, and *S*. Typhimurium. Progress toward vaccines against iNTS disease has been slow, despite its substantial global disease burden. Thus, there are high interests in exploring vaccines capable of providing protection against major pathogenic serovars.

Glycoconjugate vaccines are among the most advanced class of next‐generation *Salmonella* vaccine candidates and offer clear advantages over unconjugated polysaccharide vaccines [16]. As a Gram‐negative bacterium, *Salmonella* abundantly expresses lipopolysaccharide (LPS) on its surface. LPS is both a virulence factor and a target for protective antibodies. Structurally, LPS consists of lipid A (the endotoxin anchor in the outer membrane), a conserved core oligosaccharide bridging lipid A with an O‐polysaccharide (OPS), which can have repeating units ranging from 2 to over 100 units [17, 18] with 67 distinct OPS sequences distinguishing various serotypes. Current OPS based vaccine candidates are generally only effective against the specific serovar that the OPS is isolated from, and show little cross‐protection against other pathogenic serovars [19, 20, 21]. The availability of a single antigen based broad‐spectrum vaccine, which would obviate the need to manufacture and administer multiple vaccine constructs, would be a gamechanger for *Salmonella* vaccine design.

The vast majority of invasive human *Salmonella* isolates belong to three serogroups (A, B, and D), with groups B and D accounting for 90% of NTS isolates obtained from normally sterile sites [22]. These serogroups share a conserved trisaccharide backbone [→2)‐D‐mannose (Man*p*)‐α‐(1→4)‐ L‐rhamnose (Rha*p*)‐α‐(1→3)‐D‐galactose (Gal*p*)‐α‐(1→] in each repeating unit of their OPS [23]. On the 3‐*O* position of the mannose, a unique dideoxy hexose is attached, which defines the specific serotypes of *Salmonella*. For example, *S*. Paratyphi A has paratose (conferring the O:2 specificity, serotype A), *S*. Typhimurium has abequose (conferring the O:4 specificity, serotype B), and *S*. Enteritidis and *S*. Typhi have tyvelose (conferring the O:9 specificity, serotype D). In addition, a glucose moiety can be attached to 4‐*O* or 6‐*O* position of the galactose. To overcome the challenges of current vaccines that are generally strain‐specific, we have begun to explore the backbone of OPS as a potentially broadly protective antigen. We previously investigated the conserved OPS fragment of tri‐, hexa‐, and nona‐saccharide corresponding to one to three repeating units of the backbone [24]. Herein, we report that conjugates of such OPS backbone failed to induce protection against *S*. Paratyphi A and *S*. Typhi, two major typhoidal serovars. Surprisingly, introduction of *O*‐acetylation to the rhamnose unit within the trisaccharide antigen has been found to substantially broaden the scope of strains that can be protected. Immune sera from diacetylated trisaccharide antigen immunized rabbits provided significant protection against lethal challenges by all four major pathogenic *Salmonella* serovars in mice.

## 2 Results and Discussion

### 2.1 mQβ‐glycan 1 Conjugate Failed to Provide Protection Against *S*. Typhi and *S*. Paratyphi A

**FIGURE 1:**
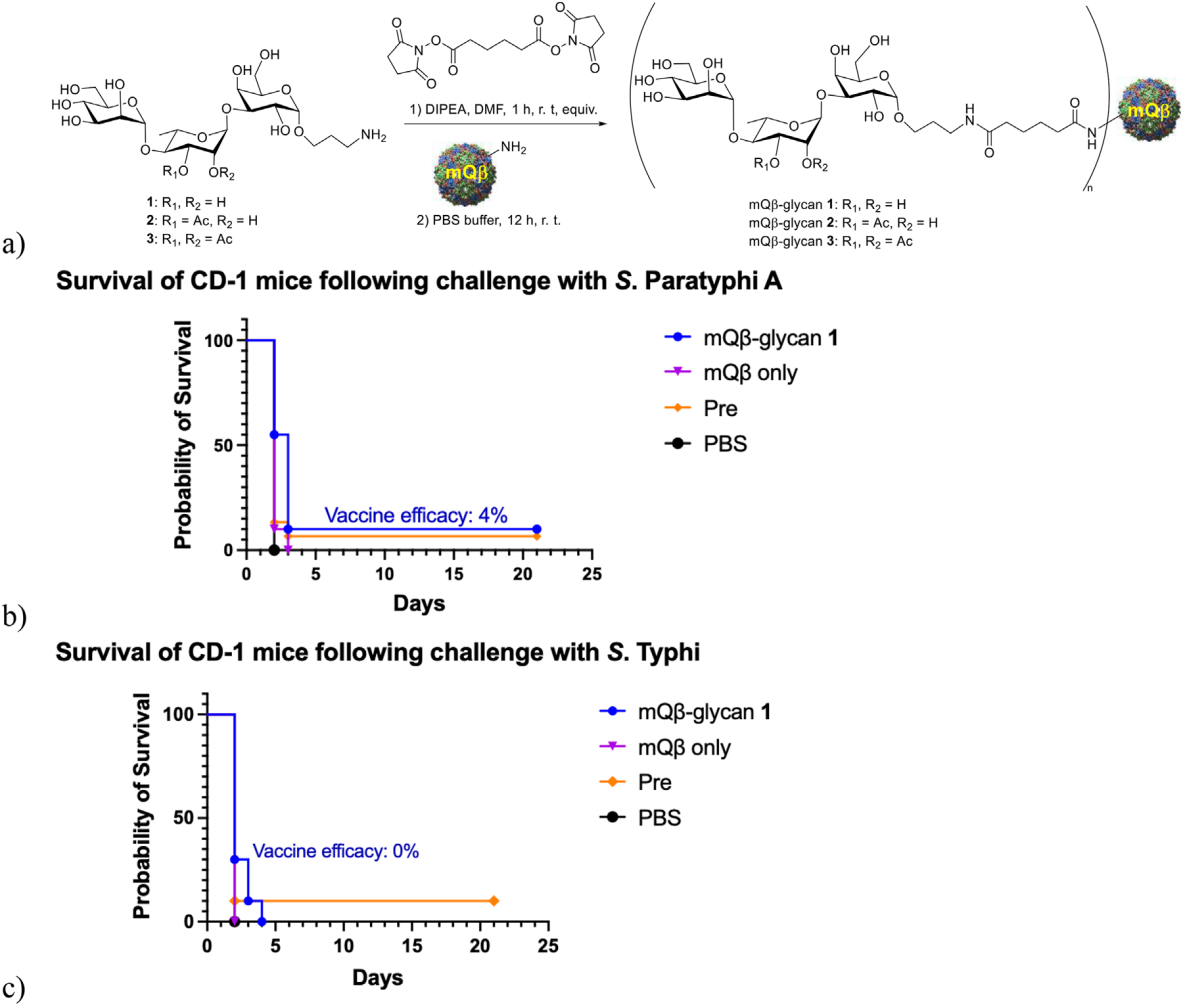
The conjugate of unacetylated trisaccharide glycan 1 with mQβ failed to provide any significant protection against *S*. Paratyphi A and *S*. Typhi infections. (a) Structures of glycans 1, 2, and 3 and scheme for mQβ glycan conjugate preparation. Naïve CD1 mice were passively administered pooled antisera from mQβ‐glycan 1‐immunized rabbits and challenged intraperitoneally with lethal doses of (b) *S*. Paratyphi A ATCC 9150 (challenge dose: 1 × 10^6^ Colony‐Forming Unit (CFU); antisera dilution: 1:10; n = 20/group) or (c) *S*. Typhi Ty2 (challenge dose: 2.5 × 10^4^ CFU; antisera dilution: 1:10; n = 10/group). VE was calculated relative to the Pre sera group as the following: VE = (1‐mortality rate of the vaccinated group/mortality rate of Pre group) × 100%. Other control groups include PBS and sera from mQβ immunzied rabbits (mQβ only).

Previously, we synthesized *Salmonella* OPS trisaccharide **1** and conjugated it with a mutant bacteriophage Qβ (mQβ) carrier (Figure 1a), which was found to protect against *S*. Enteritidis and *S*. Typhimurium following vaccination [24]. In the current study, we tested whether the mQβ‐trisaccharide **1** vaccine was effective against challenges with *S*. Paratyphi A and *S*. Typhi, as these are the major pathogenic typhoidal serovars. Naïve CD‐1 mice were administered with post‐vaccination sera from rabbits immunized with the mQβ‐trisaccharide **1** conjugate vaccine. Control groups of mice received anti‐sera from rabbits immunized with mQβ only, pre‐immune (Pre) sera, or phosphate buffered saline (PBS). All mice were then challenged with lethal doses of *S*. Typhi Ty2 or *S*. Paratyphi A ATCC 9150. Despite the fact that these strains contain the Man*p*‐Rha*p*‐Gal*p* trisaccharide backbone in their OPSs, the mQβ‐glycan **1** post‐vaccination sera did not provide any significant benefits against bacterial challenge (Figure 1b,c). This setback prompted us to re‐design the vaccine antigen.

In nature, bacterial O‐polysaccharides can be modified by *O*‐acetylation during biosynthesis. For *Salmonella*, the *O*‐acetyltransferase gtrC2 transfers an *O*‐acetyl group to 2‐*O* or 3‐*O* positions of rhamnose. These two forms may also undergo migration [25, 26], resulting in a microheterogeneous population of OPS structures. *O*‐Acetylation of rhamnose residues in OPS varies substantially among *Salmonella* serovars and across strains [25, 26, 27, 28, 29]. In *S*. Enteritidis, *O*‐acetylation of OPS is generally lower with overall *O*‐acetylation levels estimated to range from ∼10%–35% [30]. The sites of acetylation remain unclear with ∼9% reported on rhamnose [29]. *S*. Typhimurium exhibits a wide range of degree of *O*‐acetylation, from non‐detectable to high levels (∼78%–87%), with 2‐*O*‐ or 3‐*O*‐acetyl substitution observed on rhamnose [31, 32]. *S*. Typhi shows ∼67% *O*‐acetylation on rhamnose [33], whereas *S*. Paratyphi A is *O*‐acetylated in most reported strains (>60%), with *O*‐acetylation on either 2‐*O* or 3‐*O* position of rhamnose [25, 34, 35].

We hypothesize that OPS *O*‐acetylation on rhamnose may impact the protective immunity. In order to probe this, we utilized chemical synthesis to produce structurally homogeneous backbone antigens bearing defined *O*‐acetylation. Besides the aforementioned non‐acetylated trisaccharide **1**, we designed two additional OPS trisaccharides **2** and **3** incorporating (i) 3‐*O*‐acetyl group, and (ii) two *O*‐acetyl groups introduced at both 2‐*O* and 3‐*O* positions of rhamnose respectively (Figure 1a). Only the 3‐*O*‐acetylated rhamnose variant was synthesized rather than separate 2‐*O* and 3‐*O*‐acetylated forms since the *O*‐acetyl group may undergo intramolecular migration [36].

### 2.2 Convergent Stereoselective Synthesis of Acetylated *Salmonella* OPS Associated Glycans 2 and 3

**SCHEME 1:**
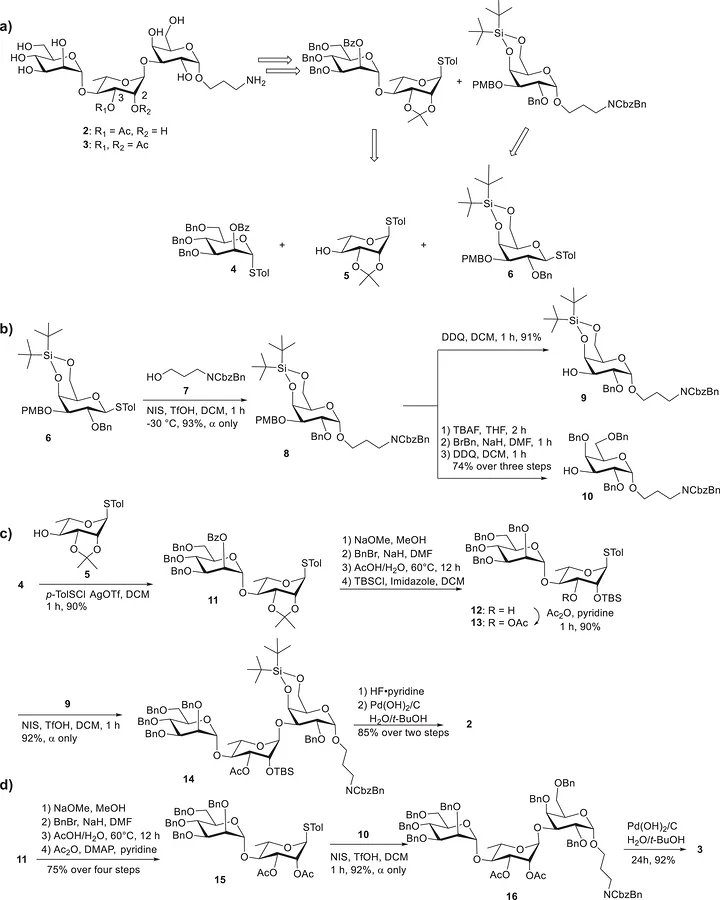
(a) Retrosynthetic analysis of *Salmonella* OPS like glycans **2** and **3**. Syntheses of (b) monosaccharide building blocks **9** and **10**; (c) the monoacetylated trisaccharide antigen **2**; and (d) the diacetylated trisaccharide antigen **3**.

It was envisaged that the target trisaccharides **2** and **3** can be obtained by stereoselective glycosylation of a common set of monosaccharide intermediates **4**, **5**, and **6** (Scheme 1a) followed by divergent modification post‐glycosylations. The *O*‐2 benzoyl (Bz) group in **4** and the *O*‐4,6 silylidene group in **6** were employed to facilitate the formation of α glycosidic linkages. The synthesis commenced from the stereoselective 1,2‐*cis* glycosylation of the silylidene [37] bearing thioglycoside derivative **6** with 3‐*N*‐(benzyl) benzyloxycarbonyl‐amino‐1‐propanol **7** (Scheme 1b). This reaction was promoted by *N*‐iodosuccinimide (NIS) and triflic acid (TfOH) affording compound **8** in 93% yield. Subsequent removal of the *p*‐methoxybenzyl (PMB) protecting group furnished compound **9** in 91% yield. Transformation of compound **8** into the monosaccharide acceptor **10** involved the deprotection of the silylidene, benzylation, and subsequent PMB removal, affording **10** in 74% overall yield (Scheme 1b).

The non‐reducing end disaccharide donor for **2** was prepared by preactivation [38] of thiomannosyl donor **4** with *p*‐tolylsulfenyl chloride (*p*‐TolSCl)/AgOTf, followed by glycosylation with **5**, affording disaccharide **11** in 90% yield (Scheme 1c). Subsequent replacement of the *O*‐2 Bz group of the mannose residue with a benzyl (Bn) group, followed by removal of the rhamnose isopropylidene group and selective protection of the C‐2 hydroxyl group with a *t*‐butyldimethyl silyl (TBS) moiety, afforded compound **12**. Transformation of **12** into the disaccharide donor **13** was accomplished by acetylation of the C‐3 hydroxyl group of the rhamnose residue. The position of the acetyl group was confirmed by a distinct long‐range ^1^H‐^13^C correlation between the rhamnose C‐3 carbon (73.09 ppm) and the methyl protons of the acetyl group (2.06 ppm) in the Heteronuclear Multiple Bond Correlation (HMBC) nuclear magnetic resonance (NMR) spectrum. A [2+1] glycosylation between **13** and **9** afforded the protected trisaccharide **14** as a single isomer in 92% yield (Scheme 1c). Deprotection of **14** was carried out by the removal of silyl ether groups, followed by hydrogenolysis providing trisaccharide **2** in 85% overall yield. The glycosidic configurations of **2** were unambiguously established by NMR analysis of the ^3^ *J*_H‐1/C‐1_ coupling constants [39]. ^3^ *J*_H‐1/C‐1_ values of 172.3 Hz were found for both the α‐Glc*p* and α‐Rha*p* linkages, and 170.3 Hz was associated with the α‐Man*p* supporting all α linkages within this trisaccharide.

In order to prepare the diacetylated trisaccharide **3**, disaccharide donor **11** (Scheme 1d) was converted into donor **15** by replacing the C‐2 Bz group with a Bn group, followed by substitution of the isopropylidene protecting group with two acetyl groups. Subsequent reaction of **15** with monosaccharide **10**, promoted by NIS/TfOH, proceeded with excellent stereoselectivity to give compound **16** in 92% yield. Global deprotection of **16** by hydrogenolysis furnished trisaccharide **3**. All glycosidic bond configurations in **3** were α as verified by NMR data (^3^ *J*_H‐1/C‐1_ coupling constants): [39] 172.3 Hz corresponds to α‐Glc*p* linkage, 174.2 Hz corresponds to α‐Rha*p*, and 170.3 Hz is associated with the α‐Man*p*.

### 2.3 *Salmonella* OPS Based Trisaccharides Were Successfully Conjugated With the mQβ Carrier to Enable Vaccine Studies

Since carbohydrate antigens alone are generally unable to activate helper T cells that are essential for the induction of high‐affinity, long‐lasting anti‐glycan IgG responses, their conjugation to an immunogenic carrier protein is indispensable. Bacteriophage mQβ serves as a powerful carrier platform for glycoconjugate‐based vaccine development [40, 41, 42, 43, 44, 45], owing to its capacity to present antigens in a highly organized fashion, thereby eliciting robust antibody responses. Conjugation of trisaccharides **2** and **3** to the bacteriophage mQβ virus‐like particle was next investigated. Trisaccharides **2** and **3** were derivatized with the bifunctional linker bis‐*N*‐hydroxysuccinimide adipate **17** and subsequently incubated with bacteriophage mQβ in PBS for 12 h to form the conjugates through amide bonds with amine groups on mQβ (Scheme S2). To establish the impacts of acetylation and directly compare the performance of the trisaccharide antigens, the synthesis of conjugate of glycan **1** with mQβ was repeated. Mass spectrometry (MS) analysis of the mQβ conjugates confirmed high levels of glycan display: mQβ‐glycan **1** conjugate bore an average of 635 glycans per capsid, mQβ‐glycan **2** conjugate contained an average of 498 copies of glycan per capsid, and mQβ‐glycan **3** conjugate had an average of 595 glycans per capsid (Scheme S2 and Figure S1). To facilitate enzyme linked immunosorbent assay (ELISA) of anti‐glycan antibodies produced, trisaccharides **1–3** were conjugated to bovine serum albumin (BSA) (Scheme S2 and Figure S2).

### 2.4 mQβ‐glycan Conjugates Induced High IgG Antibody Responses in Mice and Rabbits

**FIGURE 2:**
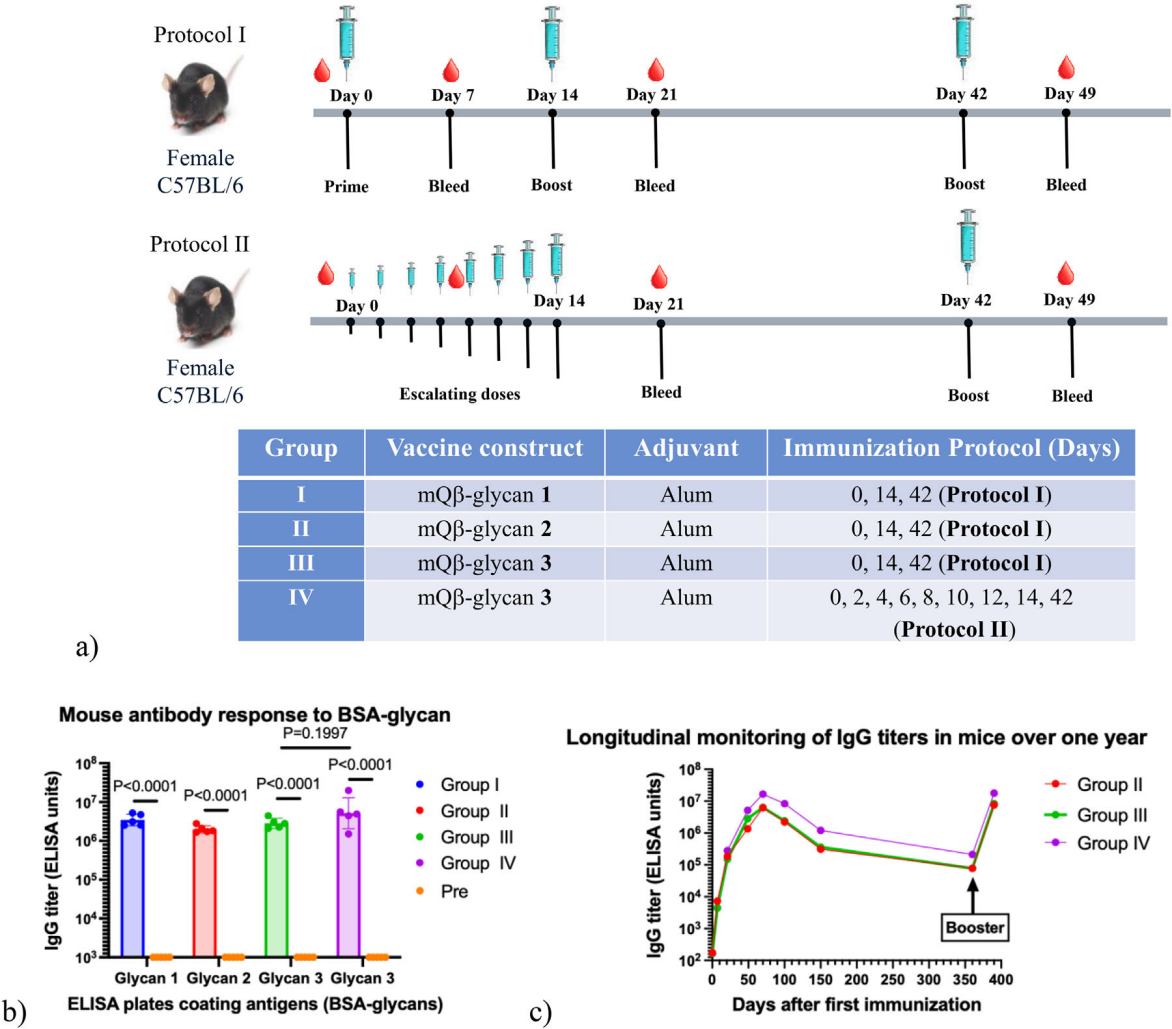
Robust serum anti‐glycan IgG antibody responses were induced by mQb‐glycans in mice. a) Mouse immunization protocols. (b) Anti‐glycan IgG titers on day 49 after the third immunization in Qβ‐glycan **1** (Group I), Qβ‐glycan **2** (Group II), and Qβ‐glycan **3** (Group III) immunized groups and in pre‐immunized mice (Pre). Each symbol represents one mouse. Geometric mean titers with the standard deviation are plotted. Statistical analysis was performed using a 2‐tailed Student's t‐test. (c) IgG titers induced by mQβ‐glycan **2** and mQβ‐glycan **3** from pooled sera of groups II‐IV monitored over 390 days.

To evaluate whether the mQβ‐glycan conjugates could elicit anti‐glycan antibody responses, groups of C57BL/6 mice were immunized subcutaneously. Aluminum hydroxide (Alum), a classical adjuvant widely used in licensed human vaccines [46], was employed for all groups. A group of mice (group I) received bolus injections (Protocol I, Figure 2a) of mQβ‐glycan **1** formulated with Alum adjuvant (15 µg of glycan **1** and 100 µg of adjuvant per injection). Groups II and III were similarly immunized with bolus injections of mQβ‐glycan **2** and mQβ‐glycan **3** conjugates, respectively (15 µg of glycan and 100 µg of adjuvant per injection). Group IV received mQβ‐glycan **3** formulated with Alum using a slow‐delivery immunization strategy termed escalating dose (Protocol II, Figure 2a). This slow delivery strategy has been shown to generate long lasting geminal centers and enhance antibody responses [47]. In this protocol, the prime and first booster doses of mQβ‐glycan **3** (30 µg of glycan **3** and 200 µg of adjuvant) were fractionated into eight gradually increasing sub‐doses, administered every other day over 14 days (Protocol II, Figure 2a). Sera were collected at baseline (day 0) and on days 7, 21, and 49. ELISA analysis demonstrated that post‐immune sera from all four groups elicited robust anti‐glycan IgG responses against the BSA‐glycan conjugates (Figure 2b). On day 49, the average IgG titers for Groups I‐IV were 3.5 × 10^6^, 2.0 × 10^6^, 2.8 × 10^6^ and 5.2 × 10^6^ ELISA units respectively, representing more than three orders of magnitude increases compared to pre‐immune sera.

Durability of the antibody response, a key attribute of effective vaccines, was evaluated next. Longitudinal monitoring over >390 days revealed that immunization with mQβ‐glycan **2** and mQβ‐glycan **3** induced highly persistent serum antibody titers. As shown in Figure 2c, IgG responses peaked around day 70, reaching 6.2 × 10^6^ (Group II) and 6.3 × 10^6^ (Group III) ELISA units. Group IV gave higher IgG titers (1.7 × 10^7^ ELISA units) than that of group III. Strong antibody levels were maintained on day 360 with IgG titers of 7.8 × 10^5^ (Group II), 7.8 × 10^5^ (Group III), and 2.1 × 10^6^ (Group IV) ELISA units. Following a booster on day 360, IgG titers rebounded to near‐peak levels, indicating that vaccine‐induced immune memory had been established and could be efficiently recalled even after prolonged intervals. Together, these findings demonstrate that mQβ‐glycan **2** and mQβ‐glycan **3** elicit potent, durable, and recallable serum IgG responses, highlighting their potential as promising vaccine candidates.

**FIGURE 3:**
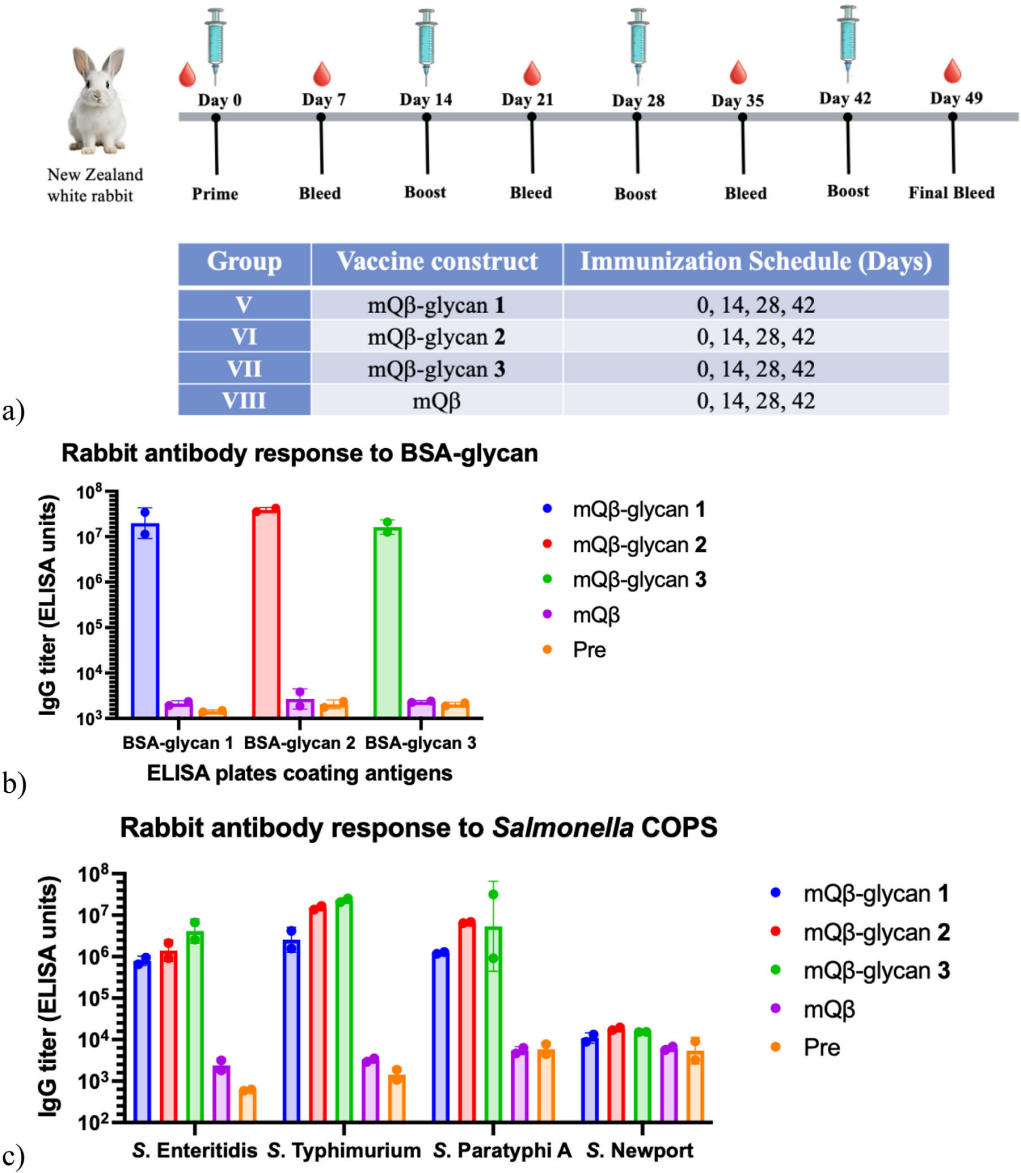
Rabbits mounted high anti‐glycan IgG titers in response to immunization with mQb‐glycan 1–3 conjugates. a) Rabbit immunization protocols. (b) Endpoint values of IgG titers against the three trisaccharide antigens **1**–**3** used for immunization. (c) ELISA analysis for antibody binding to native *Salmonella* COPS. Individual antisera from rabbits immunized with mQβ‐glycan **1**, mQβ‐glycan **2**, mQβ‐glycan **3**, and mQβ, were assessed for COPS binding by ELISA as indicated. Each symbol represents one rabbit. Data are presented as geometric mean values ± standard deviation. mQβ‐glycan **1** (day 49 sera from two rabbits immunized with mQβ‐glycan **1**); mQβ‐glycan **2** (day 49 sera from two rabbits immunized with mQβ‐glycan **2**); mQβ‐glycan **3** (day 49 sera from two rabbits immunized with mQβ‐glycan **3**); mQβ (day 49 sera from two rabbits immunized with mQβ); and Pre (pre‐immunized sera from rabbits).

To assess immunogenicity across species and facilitate translational studies, rabbits were immunized subcutaneously with mQβ‐glycan **1**–**3** conjugates. Four groups of rabbits received different vaccine as outlined in Figure 3a with injections administered on days 0, 14, 28, and 42, and the terminal blood collection on day 49. ELISA analysis using BSA‐glycans as the coating antigen revealed strong induction of antigen‐specific IgG responses in the immunized groups. Group V (mQβ‐glycan **1**) generated high levels of anti‐glycan **1** IgG antibodies (2.2 × 10^7^ ELISA units), as compared to average titer of 1.2 × 10^3^ ELISA units in pre‐immune sera (Figure 3b). Group VI (mQβ‐glycan **2**) elicited high levels of anti‐glycan **2** IgG antibodies with a geometrical mean titer of 3.8 × 10^7^ ELISA units vs 1.3 × 10^3^ ELISA units in pre‐immune sera. Group VII (mQβ‐glycan **3**) produced similarly strong anti‐glycan **3** responses (1.6 × 10^7^ ELISA units, pre‐immune sera: 1.4 × 10^3^ ELISA units). In contrast, sera from the control group VIII (mQβ alone) exhibited only baseline reactivity (IgG titers: glycan **1**, 3.4 × 10^3^ ELISA units; glycan **2**, 2.9 × 10^3^ ELISA units; glycan **3**, 2.8 × 10^3^ ELISA units). No adverse effects were observed in rabbits throughout the vaccination studies.

Besides recognition of the synthetic trisaccharide antigen, the antibodies induced should be capable of recognizing the native polysaccharide from the pathogenic *Salmonella* strains. In order to test this, we purified the core and O‐polysaccharide (COPS) from *S*. Paratyphi A, *S*. Typhimurium, and *S*. Enteritidis. The COPSs of these serovars of *Salmonella* have the same trisaccharide backbone sequence. To establish the selectivity of the antibodies, COPS from another *Salmonella* serovar, *S*. Newport, which possessed a different OPS backbone structure (O:6,9), was also isolated. Day 49 rabbit sera were analyzed in ELISA using COPS as the coating antigen. The rabbit sera exhibited markedly enhanced binding to all COPS tested (Figure 3c), including *S*. Enteritidis COPS (1303, 2309 and 6834 folds for mQβ‐glycan **1**, **2**, and **3** respectively compared to pre‐immune sera), *S*. Typhimurium COPS (1810, 10630, and 15989 folds for mQβ‐glycan **1**, **2**, and **3** respectively compared to pre‐immune sera), and *S*. Paratyphi A COPS (211, 1136 and 923 folds for mQβ‐glycan **1**, **2**, and **3** respectively compared to pre‐immune sera). Enhanced binding was not observed against *S*. Newport COPS suggesting that antibody binding was selective to the backbone shared by *S*. Enteritidis, *S*. Typhimurium, and *S*. Paratyphi A (Figure 3c). In contrast to rabbit sera, despite the high IgG titers observed against the synthetic glycans **1**–**3** in sera from mice immunized with mQβ‐glycan conjugates, mouse sera showed only minimal reactivities (<10‐fold increase compared to pre‐immune sera) toward native COPS (data not shown). As a result, the rest of the study focused on the analysis of rabbit sera.

**FIGURE 4:**
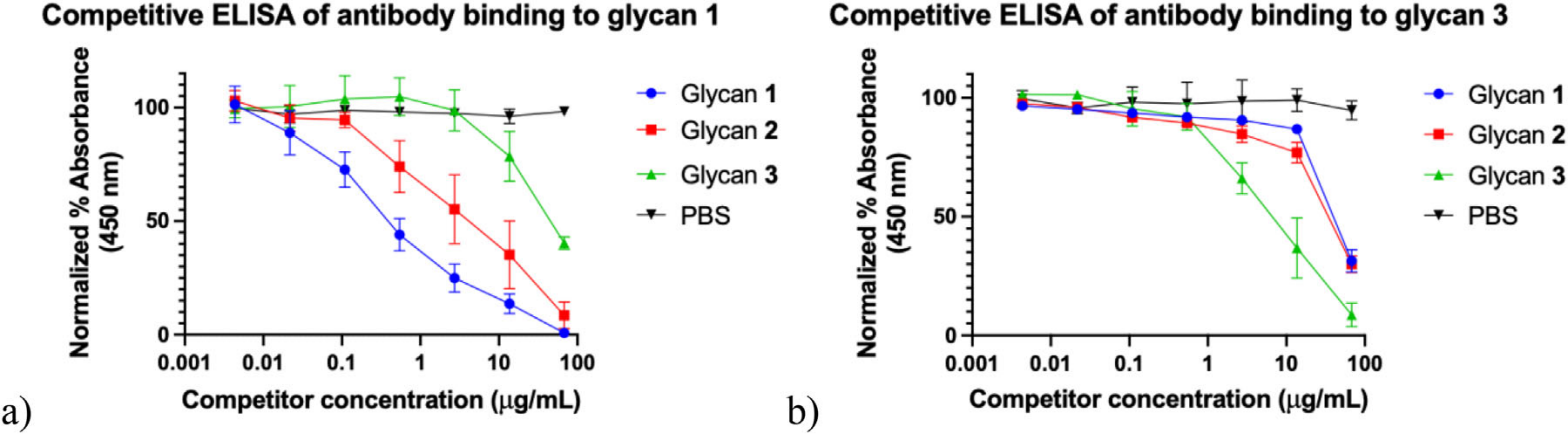
Competitive ELISA analysis of the specificity and relative affinity of anti‐glycan antibodies (two rabbits per group; each rabbit sera sample assayed in triplicate). Free glycans were used as competitors at concentrations ranging from 0.004 µg/mL to 68 µg/mL. a) Normalized binding of the BSA‐glycan **1** to IgG antibodies from D49 post‐immune sera of rabbits immunized with mQβ‐glycan **1**, in the presence of increasing concentrations of glycan **1**, glycan **2**, glycan 3, or a no‐competitor control (PBS). b) Normalized binding of the BSA‐glycan **3** to IgG antibodies from D49 post‐immune sera of rabbits immunized with mQβ‐glycan **3**, in the presence of increasing concentrations of glycan **1**, glycan **2**, glycan **3**, or a no‐competitor control (PBS).

To further characterize rabbit antibody specificity, competition ELISA was performed using BSA‐glycan **1** or BSA‐glycan **3** conjugates as coating antigens, which were then incubated with sera from mQβ‐glycan **1** (Figure 4a) or mQβ‐glycan **3** (Figure 4b) immunized rabbits respectively. Increasing concentrations of free glycans **1**–**3** were added to the wells to compete against BSA‐glycan conjugate for binding with rabbit antibodies. For anti‐mQβ‐glycan **1** sera, binding to BSA‐glycan **1** immobilized in ELISA wells was most effectively inhibited by glycan **1** (IC_50_ = 0.4 µM), followed by glycan **2** (IC_50_ = 3.1 µM), and the least by glycan **3** (IC_50_ = 26.8 µM) (Figure 4a). Conversely, for anti‐mQβ‐glycan **3** sera, inhibition was the strongest with glycan **3** (IC_50_ = 7.1 µM), whereas glycan **2** (IC_50_ = 22.1 µM) and glycan **1** (IC_50_ = 26.1 µM) exhibited much weaker inhibitory effects (Figure 4b). These results confirm that the induced antibodies preferentially recognize the glycan epitopes in the vaccine while retaining partial cross‐reactivity to glycans varying in rhamnose *O*‐acetylation patterns.

### 2.5 Post‐Immune Antisera Recognized *Salmonella* Bacteria

**FIGURE 5:**
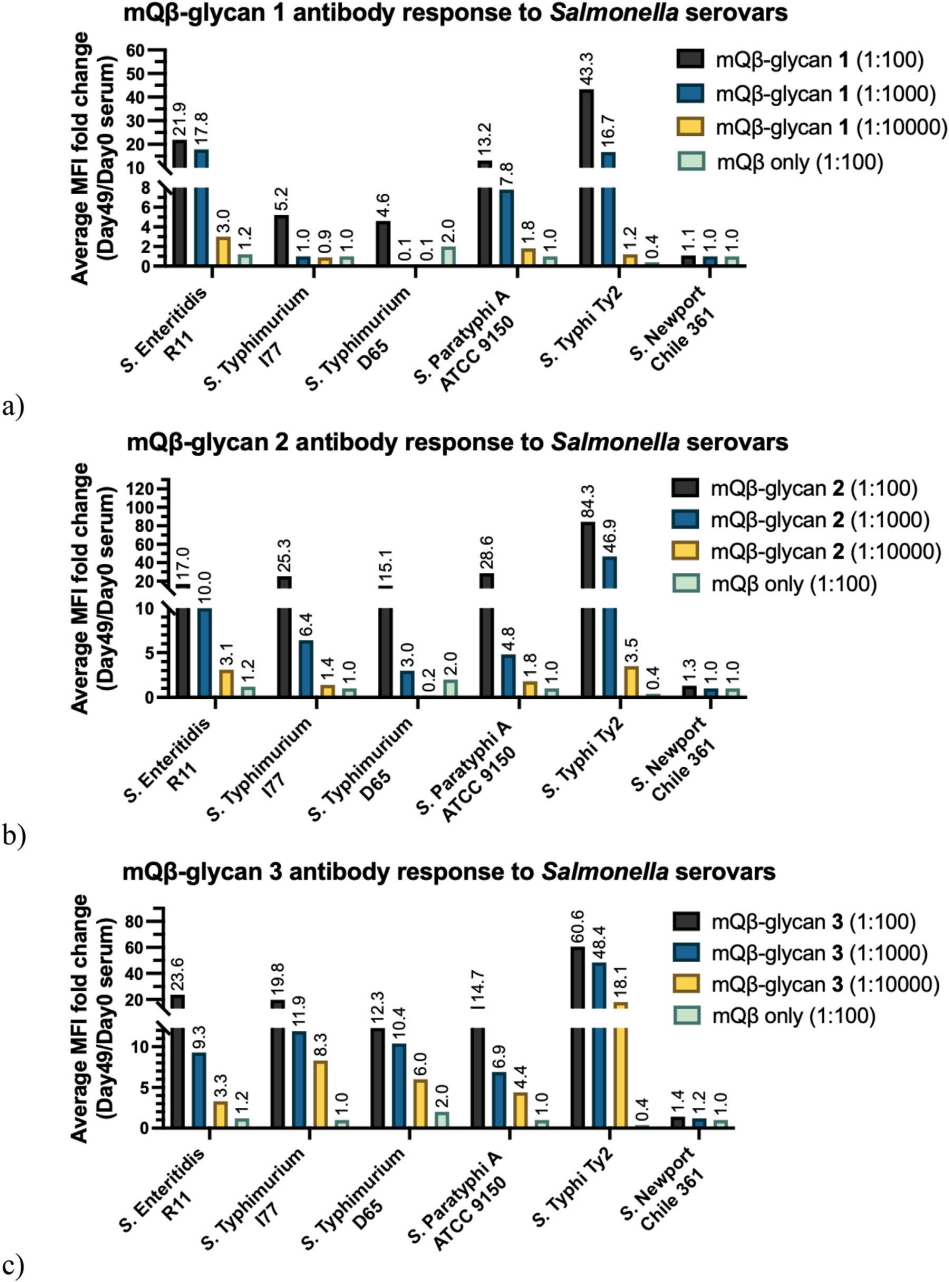
mQβ‐glycan 1, mQβ‐glycan 2 and mQβ‐glycan 3 induced rabbit antibodies recognize the surface of *Salmonella* serovars. Flow cytometry data of a) mQβ‐glycan 1; (b) mQβ‐glycan 2; and (c) mQβ‐glycan 3 post‐immunization sera (1: 100, 1:1000, 1:1000 dilution) binding with *S*. Enteritidis R11, *S*. Typhimurium I77, *S*. Typhimurium D65, *S*. Paratyphi A ATCC 9150, *S*. Typhi Ty2, *S*. Newport Chile 361 strains, as compared to the anti‐mQβ rabbit IgG (1: 100 dilution). The numbers above each column were the average fold change of median fluorescence intensity (MFI) of Day 49 sera over Day 0 sera.

The abilities of antisera to bind live *Salmonella* bacteria were next evaluated by flow cytometry. As shown in Figure 5, the binding of post‐immune and pre‐immune antisera was compared across multiple *Salmonella* serovars. Post‐immune sera from rabbits immunized with mQβ‐glycan **1**, mQβ‐glycan **2** and mQβ‐glycan **3** were tested at three dilutions to assess binding strength. For the target serovars (*S*. Enteritidis R11, *S*. Typhimurium I77, *S*. Typhimurium D65, *S*. Paratyphi A ATCC 9150, *S*. Typhi Ty2), post‐immune sera from all mQβ‐glycan immunization groups bound strongly to bacteria, displaying clear concentration‐dependent responses with serum dilutions (Figure 5). In contrast, negligible binding was detected against *S*. Newport Chile 361, consistent with the absence of cross‐reactive epitopes. Sera from the mQβ‐only control group showed little binding to all bacteria as compared with pre‐immune sera. Collectively, these results demonstrate that immunization with the glycoconjugate vaccines elicits strong and specific antibody responses recognizing the targeted *Salmonella* serovars.

To confirm the binding of antibodies with *Salmonella* bacteria observed in flow cytometry, transmission electron microscopy (TEM) was used as a qualitative approach to visualize cell surface antibody engagement. Because the objective of TEM analysis was illustrative rather than comparative across all serovars, *S*. Paratyphi A ATCC 9150 was used as a representative example. Pre‐immune and post‐immune sera from rabbits immunized with mQβ‐glycan **1**, mQβ‐glycan **2**, or mQβ‐glycan **3** were each applied to fixed *S*. Paratyphi A cells, followed by detection using ImmunoGold‐labeled goat anti‐rabbit IgG (10 nm). Pre‐immune serum showed no detectable binding of gold particles on bacteria (Figure S3a). In contrast, sera from mQβ‐glycan **1** (Figure S3b), mQβ‐glycan **2** (Figure S3c), and mQβ‐glycan **3** (Figure S3d) all exhibited clear surface labeling, supporting flow cytometry results that vaccine‐induced antibodies can bind with bacteria.

### 2.6 Antisera From mQβ‐glycan 3 Immunized Rabbits Provided Significant Protection to Mice Against Death Induced by Four Major Pathogenic *Salmonella* Serovars

Having demonstrated that antibodies elicited by the glycoconjugate vaccines robustly recognized and bound to *Salmonella* cells, we next sought to determine whether vaccine induced antibodies could confer functional protection in vivo from lethal *Salmonella* challenges. Pooled pre‐immune or post‐vaccination sera were administered into naïve mice, which was followed 5 h later by intraperitoneal challenge with *S*. Enteritidis R11, *S*. Typhimurium I77, *S*. Paratyphi A ATCC 9150 or *S*. Typhi Ty2. Survival rates were monitored over time.

**FIGURE 6:**
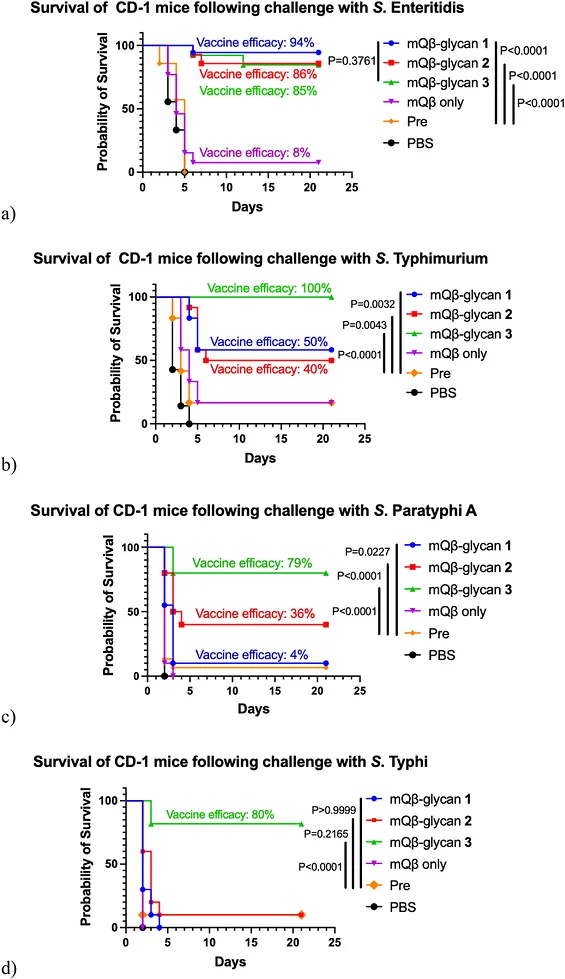
Protective effects of antisera from immunized rabbits. Naïve CD‐1 mice were passively administered pooled antisera from mQβ‐glycan‐immunized rabbits and challenged intraperitoneally with lethal doses of a) *S*. Enteritidis R11 (challenge dose: 1.3 × 10^6^ CFU; antisera dilution: 1:100; n = 18 for glycan **1** group; n = 12 for glycan **2** and glycan **3** groups); (b) *S*. Typhimurium I77 (challenge dose: 0.5 × 10^6^ CFU; antisera dilution: 1:200; n = 12/group); (c) *S*. Paratyphi A ATCC 9150 (challenge dose: 1 × 10^6^ CFU; antisera dilution: 1:10; n = 20/group); or (d) *S*. Typhi Ty2 (challenge dose: 2.5 × 10^4^ CFU; antisera dilution: 1:10; n = 10/group). VE was calculated relative to the pre‐immune sera group. Statistical analysis for survival was performed using the logrank test.

For the *S*. Enteritidis R11 challenge (Figure 6a), all mice receiving PBS or pre‐immune sera succumbed to bacterial infections, whereas those receiving mQβ immune sera showed only minimal protection (VE = 8%). In contrast, mice receiving mQβ‐glycan immune sera exhibited strong protection with statistically similar protective efficacies of 94%, 86%, and 85% for mQβ‐glycan **1**, mQβ‐glycan **2**, and mQβ‐glycan **3** respectively. For the *S*. Typhimurium I77 challenge (Figure 6b), all mice given PBS died within four days. While mQβ‐glycan **1** and mQβ‐glycan **2** sera provided partial protection (VE = 50% and 40%, respectively), mQβ‐glycan **3** immune sera conferred complete protection (VE = 100%). For the *S*. Paratyphi A ATCC 9150 challenge, as shown in Figure 6c, all mice receiving PBS sera or mQβ sera died and only marginal protection was observed with mQβ‐glycan **1** sera (VE = 4%). In contrast, mQβ‐glycan **3** sera conferred significant protection (VE = 79%) (Figure 6c). For *S*. Typhi Ty2 challenge, while PBS, mQβ sera recipients or those receiving mQβ‐glycan **1** sera all died by day 4, administration of mQβ‐glycan **3** sera provided strong protection with a VE of 80% (Figure 6d). Therefore, excingtly, sera generated by mQβ‐glycan **3** immunization provided significant protection against lethal challenges by all four major pathogenic serovars of *Salmonella*.

At 22 days post‐challenge, spleens and kidneys from all surviving mice were collected to assess bacterial persistence. No colonies were found from any organs of any surviving animal, indicating complete bacterial eradication. (As all CFU values were zero, these results are reported textually without a corresponding figure).

### 2.7 Immunization of Diacetylated Trisaccharide Based Vaccine Led to Significant Enhancement of Complement Deposition and Opsonophagocytosis

To better understand the superior protection bestowed by the diacetylated trisaccharide based vaccine, in vitro assays were performed. While antigen recognition through the fragment antigen binding region (Fab) is essential for antibody‐mediated immunity, effective microbial clearance can depend on effector mechanisms mediated by the fragment crystallizable (Fc) domain [48]. Two major Fc‐dependent mechanisms are complement activation and phagocyte engagement through Fc receptors [49].

**FIGURE 7:**
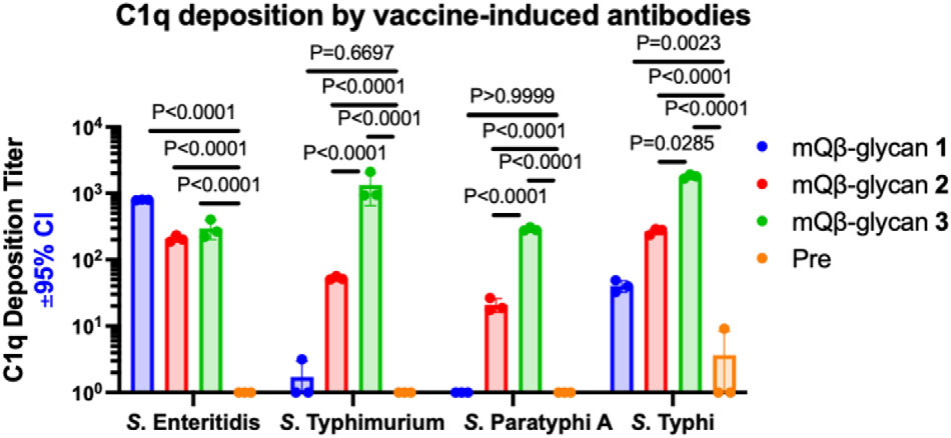
Deposition of C1q onto four serovars of *Salmonella* were observed mediated by sera from rabbits immunized with mQβ‐glycan 1, glycan 2 and glycan 3 conjugates. Complement deposition tests were performed as described [51] using pooled sera from rabbits (n = 2 per group) immunized with mQβ‐glycan **1**, glycan **2** and glycan **3** conjugates. Titers and 95% confidence intervals (CI) were determined by linear regression using log10 values of the serum dilutions to determine the X intercept and 95% CI when Y = 0.25 (OD_405_ of ELISA plate reading). Three aliquots were prepared from each pooled serum, and the individual values of the three aliquots were presented. Pre: pre‐immunization mouse sera; *S*. Enteritidis: *S*. Enteritidis R11; *S*. Typhimurium: *S*. Typhimurium I77; *S*. Paratyphi A: *S*. Paratyphi A ATCC 9150; *S*. Typhi: *S*. Typhi Ty2. Data are presented as geometric mean values ± standard deviation. Statistical significance was determined using one‐way ANOVA followed by Tukey's multiple comparisons test.

Activation of the classical complement pathway is initiated by C1q recognition of antigen‐bound antibodies, and efficient C1q engagement requires clustered Fc domains generated through antigen‐driven IgG hexamerization [50, 51]. To evaluate antibody‐mediated complement recruitment, we incubated immobilized *Salmonella* strains with pooled immune rabbit sera alongside IgM‐depleted 2.5% rabbit complement (Figure 7). Following incubation, the detection of deposited C1q was performed using anti‐C1q antibodies. As shown in Figure 7, sera from mQβ‐glycan **3** group induced significantly greater C1q deposition than pre‐immune sera across all four *Salmonella* strains. Moreover, mQβ‐glycan **3** elicited markedly higher C1q binding than mQβ‐glycan **1** and mQβ‐glycan **2** in *S*. Typhimurium I77, *S*. Paratyphi A ATCC 9150 and *S*. Typhi Ty2. In comparison, although anti‐mQβ‐glycan **1** sera bound to all four strains by ELISA (Figure 3a), it did not promote detectable C1q deposition on *S*. Typhimurium and *S*. Paratyphi A relative to the pre‐immune serum. These findings indicate that antibody binding alone is insufficient for effective complement engagement and suggest that glycan **3** elicits antibodies with enhanced capacity to support Fc clustering and productive C1q recruitment on the bacterial surface.

**FIGURE 8:**
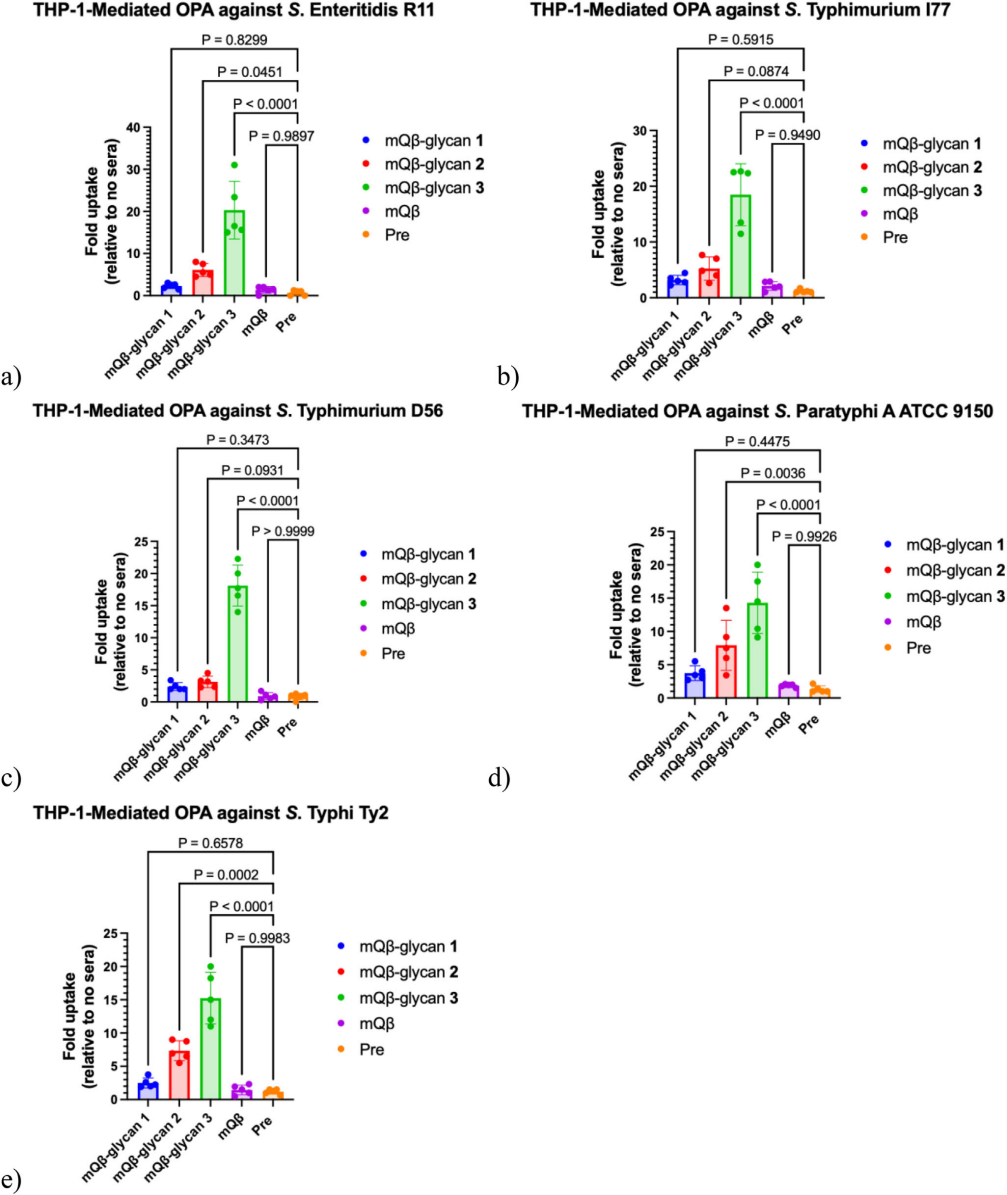
OPA of five *Salmonella* strains by THP‐1‐derived human macrophages. Five *Salmonella* strains were incubated with pooled rabbit sera (Pre, mQβ, mQβ‐glycan **1**, mQβ‐glycan **2** or mQβ‐glycan **3** immune sera) diluted 1:5, followed by incubation with differentiated THP‐1. Opsonophagocytic uptake is presented as fold uptake relative to the media only (no serum) control. Each data point represents one technical replicate (four replicates total per condition). Data are presented as geometric mean values ± standard deviation. Statistical significance was determined using one‐way ANOVA followed by Tukey's multiple comparisons test.

Besides complement deposition, a complement independent mechanism of immune protection is opsonophagocytic uptake of bacteria upon antibody binding. Opsonization with subsequent phagocytic internalization by macrophages constitutes a major mechanism by which antibodies contribute to the elimination of *Salmonella*. We next performed opsonophagocytic uptake assays (OPA) with human THP‐1‐derived macrophages on five *Salmonella* strains, i.e., *S*. Enteritidis R11, *S*. Typhimurium D65, *S*. Typhimurium I77, *S*. Paratyphi A and *S*. Typhi Ty2. The bacteria were incubated with pre‐immune, mQβ‐only rabbit sera, or sera from rabbits immunized with mQβ‐glycan **1**–**3** conjugates. Pre‐immune and mQβ immunized rabbit sera did not promote significant bacterial uptake compared with media controls (no sera). In contrast, anti‐mQβ‐glycan **3** sera markedly enhanced opsonization of all five tested *Salmonella* strains by macrophages (Figure 8), which supported significant in vivo protection observed against all serovars by these sera (Figure 6).

### 2.8 Computational Assessment of *O*‐Acetylation Effects on Conformation of Trisaccharide Antigens

With the substantially different protective efficacy observed between mQβ conjugates with glycan **1** vs glycan **3,** we sought to better understand the structural differences of the two glycan antigens. Density functional theory (DFT) calculations with B3LYP functional [52, 53] and the 6–311++G(d,p) basis set were performed to probe the accessible conformational space of trisaccharides **1** and **3**. Analysis of the DFT‐optimized structures revealed that both structures are primarily in an extended conformation. The *O*‐acetyl groups in trisaccharide **3** project outward from the glycan backbone (Figure S4). Superposition of structures of glycan **3** and glycan **1** showed that the conformations of the two molecules are similar (Figure S5) suggesting *O*‐acetylation does not substantially perturb the overall conformation.

### 2.9 Analysis of Structures of Trisaccharide Antigens Under Bioconjugation Conditions

The *O*‐acetyl moiety in a carbohydrate may undergo intramolecular migration between various hydroxyl groups [36] or get cleaved in vitro or in vivo. As it is challenging to determine whether the *O*‐acetyl groups on mQβ‐glycan **2** or mQβ‐glycan **3** remained intact in animals, in vitro analyses were performed. Mass spectrometry results (Figure S1) demonstrated that the molecular weights of acetylated glycans did not change after bioconjugation, suggesting they retained the *O*‐acetyl groups in the conjugation process. To test the possibility of intramolecular migration of *O*‐acetyl, glycans **2** and **3** were incubated in PBS buffer at pH 7.4 (the condition used for mQβ conjugation) for four days. NMR analysis of the samples showed that for glycan **2** containing a 3‐*O*‐acetyl group, a new set of NMR signals appeared (Figure S6a), which was consistent with the structure of compound **S1** with the migration of the *O*‐acetyl to 2‐*O* position of the rhamnose. After 12 h of incubation, the ratio of newly formed **S1** to glycan **2** reached approximately 2:3 (Figure S6a). No significant changes were observed after 12 h. In contrast, glycan **3** was found stable under this condition with no significant changes in four days (Figure S6b). The migration of *O*‐acetyl in glycan **2** is likely attributable to the presence of adjacent free hydroxyl group at C‐2 position of the rhamnose. Glycan **3** lacks free hydroxyl groups neighboring the acetyl groups, leading to its observed stability in vitro.

## 3 Discussion

*Salmonella* infections cause significant morbility and mortality worldwide [1]. As the prevelance of anti‐microbial resistant strains increases, vaccines are useful tools to reduce the rate of *Salmonella* infections and the spread of anti‐microbial resistence. A challenge for effective *Salmonella* vaccines is that there are multiple pathogenic serotypes to humans with the four major ones, i.e., *S*. Paratyphi A, *S*. Typhimurium, *S*. Typhi, and *S*. Enteritidis, belonging to distinct serogroups (A, B, and D). The surface‐exposed OPS is a potential protective antigen. Individual OPS based vaccines have been reported that utilize OPS isolated from *S*. Paratyphi A [54], *S*. Enteritidis [54], and *S*. Typhimurium [55] as antigens, or using synthetic tetrasaccharides from *S*. Enteritidis and *S*. Paratyphi A, composed of tyvelose and paratose attached to the trisaccharide backbone respectively [19, 20]. Such vaccines are generally serotype‐specific, since vaccine‐induced antibodies largely recognize strains from the serogroup bearing the same OPS structures [21]. This is due to the immuno‐dominance of the dideoxy glycan attached to the OPS backbone. To cover the multiple *Salmonella* serovars responsible for human infection, the prevaling strategy has been to prepare multiple vaccine constructs each against one serovar and mix these constructs into a combination vaccine [21, 56, 57]. This increases the barriers for manufacturing and accessibility of the vaccine.

Rather than developing multiple vaccines, we aim to significantly simplify vaccine design by targeting a single antigen for the development of a broad‐spectrum vaccine against *Salmonella*. Our earlier approach involved targeting the conserved cores of LPS shared among all *Salmonella* strains [58]. However, because the core is normally masked by the overlying OPS, this vaccine needs to be combined with a LPS transport inhibitor to be effective, limiting its utility in vivo. This has prompted us to focus on the OPS backbone.

Our new approach reported here targeted the OPS backbone trisaccharides **1**–**3**, which were synthesized stereoselectively and conjugated with mQβ. All three conjugates induced high levels of anti‐glycan IgG antibodies upon rabbit immunization and the antibodies selectively bound *Salmonella* bacteria from A, B and D groups. Anti‐sera generated against non‐acetylated glycan **1** provided high protection (VE 94%) against *S*. Enteritidis, medium protection (VE 50%) against *S*. Typhimurium, and no protections against *S*. Paratyphi A (VE 4%) or *S*. Typhi (VE 0%). In comparison, sera from diacetylated glycan **3** immunization improved the VE to *S*. Typhimurium to 100% and provided significant protection against *S*. Paratyphi A and *S*. Typhi with VE of 79% and 80% respectively, while maintaining high protection against *S*. Enteritidis. in vitro functional assays revealed that di‐*O*‐acetylation of the trisaccharide antigen (glycan **3**) led to significant enhancement of both complement deposition and opsonophagocytic uptake of bacteria by the post‐immune sera. The detailed mechanism for the superior protection in vivo bestowed by antigen acetylation will need further investigation and analyses of complex immunological factors, which may include the recruitment and activation of phagocytes, local inflammatory cues, complement‐Fc synergy, and serovar‐associated tissue tropism.

The significant enhancement of protective efficacy against *Salmonella* bestowed by *O*‐acetylation is intriguing. *O*‐Acetylation can affect properties of microbial polysaccharides [28, 29, 59]. In nature, OPS acetylation can be an immune evasion mechanism, contributing to bacterial persistence in a host [33]. For *Salmonella* vaccine design, previous studies compared OPS antigens before and after de‐*O*‐acetylation with mixed conclusions on the influence of *O*‐acetylation on the immunogenicity of OPS [29, 31, 34, 55]. In one study, after immunization with highly *O*‐acetylated‐rhamnose antigens such as heat‐inactivated bacteria or purified LPS, antibodies against *O*‐acetyl‐rhamnose were rarely detectable in immunized rabbits [60]. On the other hand, hydrazine treatment of OPS from *S*. Paratyphi A, which would remove the *O*‐acetates, led to antibodies with no bactericidal activities [34]. Utilization of Generalized Modules for Membrane Antigens (GMMA) generated from *Salmonella* bacteria for vaccination showed that *O*‐acetylation and glucosylation of OPS may help enhance serum bactericidal activity in cross‐reactivity against heterologous strains of *Salmonella* [29]. With the complex structures including secondary modifications such as *O*‐acetylation and glucosylation of OPS, and the uncertainty of complete removal of the *O*‐acetates from OPS, it is challenging to pinpoint the exact protective epitopes with naturally isolated OPS from bacteria.

The availability of a structurally well‐defined synthetic *Salmonella* trisaccharide enabled us to probe the effects of *O*‐acetylation of rhamnose of *Salmonella* OPS trisaccharide in detail. While we observed intramolecular migration of *O*‐acetyl of glycan **2** under the condition used for mQβ conjugation by NMR, glycan **3** was found to be stable in vitro (Figure S6). Due to hydrolytic instability of esters to esterases [61], it is possible that some *O*‐acetates may be removed from mQβ‐glycan **3** conjugate once administered in vivo. Partial de‐*O*‐acetylation may allow the di‐*O*‐acetylated conjugate to display a set of structurally related epitopes, including di‐*O*‐acetylated, mono‐*O*‐acetylated, and non‐acetylated forms. Such epitope diversification could potentially broaden antibody recognition and contribute to the enhanced cross‐reactive response. On the other hand, the *O*‐acetate should be at least partially retained in vivo for antibody production due to the better binding of sera obtained from mQβ‐glycan **3** conjugate immunized rabbits to diacetylated trisaccharide **3** than the non‐acetylated trisaccharide **1** and vice versa (Figure 4).

The protective efficacy of the vaccine is not simply due to differential degrees of *O*‐acetylation of OPS in various *Salmonella* serovars. OPS from *S*. Enteritidis bears little *O*‐acetylation on rhamnose [29]. *S*. Typhimurium, *S*. Paratyphi A, and *S*. Typhi exhibit higher levels with variable 2‐*O*‐ and 3‐*O*‐acetyl substitution of rahmnoses in their OPS [25]. Consistent with these observations, quantitative analyses of COPS utilized in this study revealed minimal *O*‐acetylation in *S*. Enteritidis by NMR spectroscopy, whereas *S*. Paratyphi A and *S*. Typhimurium exhibited substantially higher levels (>60% and >90%, respectively) (Figure S7). The divergent outcomes of glycan **1**–**3** based antisera in protection against lethal challenges do not correlate with differences in *O*‐acetylation levels of the four serovars. These results suggest that the enhanced activity of the di‐*O*‐acetylated trisaccharide conjugate is not merely a reflection of bulk OPS *O*‐acetylation abundance, but may arise from the specific way antibodies elicited by this defined epitope interact with OPS surfaces across different serovars.

The di‐acetylated trisaccharide identified through this study is a novel epitope, which may be present in native OPS at low concentrations, as structural studies with bacterial‐derived OPS have identified only either 2‐*O* or 3‐*O* acetate modifications [25]. O‐Acetylation is a new approach to produce antibodies capable of providing broad‐spectrum protection against multiple serovars of *Salmonella*. Previous *in‐silico* conformational analyses showed that *O*‐acetylation does not substantially alter the overall conformational landscape of *S*. Typhimurium OPS repeat units carrying different side‐chain substitutions [29, 55]. Our Density Functional Theory (DFT) calculations with a trisaccharide backbone are consistent with these findings. Compared to glycan **1**, glycan **3** has similar conformations with both *O*‐acetyl groups highly exposed along the trisaccharide backbone (Figures S4 and S5). This positions *O*‐acetates as readily accessible epitopes capable of engaging B cell receptors and antibody paratopes efficiently. Such binding may also affect the orientation of the Fc region after bacterial opsonization, ultimately modulating downstream effector functions such as complement activation and Fc receptor (FcR) engagement. Studies are underway to generate monoclonal antibodies against the di‐acetylated trisaccharide **3** in order to further understand the roles of 2‐*O*/3‐*O* acetates in immune protection.

Besides *O*‐acetylation, OPSs isolated from bacteria may contain other modifications such as glucosylation on the Gal*p* unit. The strains of *Salmonella* we studied, i.e., *S*. Enteritidis R11, *S*. Typhimurium I77, *S*. Paratyphi A ATCC 9150, and *S*. Typhi Ty2, have reported degrees of glucosylation of 25%, 12%, 57%, and 62% respectively [33, 35, 55]. The glucosylated form of O‐antigen has been shown to decrease complement binding and mediate enhanced survival of *Salmonella* in human serum [33]. Although glycan antigen **3** does not contain the glucose modification, the antisera generated by mQβ‐glycan **3** were able to protect against these serovars. On the other hand, further studies of the vaccine design can explore the incorporation of the glucose moiety into the glycan antigen to enhance vaccine efficacy.

In the current study, in vivo protection was evaluated using passive transfer of rabbit sera, which allowed us to directly assess that antibodies elicited by the synthetic trisaccharide conjugates were sufficient to mediate protection. While rabbit sera enabled effective protection, it is interesting to note that sera from mice immunized with the same vaccine constructs showed weak binding to COPS and intact *Salmonella* bacteria, suggesting that they may have limited protective activity. Similar species‐specific vaccine outcomes have been observed in previous studies [19, 20, 58, 62], likely reflecting species differences in evolutionary lineage and antibody repertoire. Future work is needed to evaluate the vaccine in larger animal models and in humans to establish its protective efficacy in the active immunization protection model.

## 4 Conclusions

Although *O*‐acetylation is a common structural feature of bacterial polysaccharides, its immunological contributions in synthetic carbohydrate vaccine design remain poorly understood. In order to develop a single antigen based broad‐spectrum vaccine against *Salmonella*, we synthesized OPS backbone based trisaccharide antigens **1**–**3**, which have the same backbone glycan sequences but differ in the number and locations of *O*‐acetates on the rhamnose moiety in the trisaccharide. The availability of these structurally well‐defined trisaccharides enabled us to probe the effects of rhamnose *O*‐acetylation on antibody responses against *Salmonella*. Interestingly, we discovered that the *O*‐acetates have a profound impact on the breadth of protection bestowed by the vaccine. While the conjugates of mQβ carrier with all three glycan antigens elicited high levels of IgG antibodies against the COPS isolated from *S*. Enteritidis, *S*. Typhimurium and *S*. Paratyphi A, the antisera from unacetylated glycan **1** immunized rabbits were ineffective in protecting against the lethal challenges by *S*. Paratyphi A and *S*. Typhi. In contrast, di‐*O*‐acetylation of the trisaccharide antigen (glycan **3**) significantly enhanced complement deposition and opsophagocytic uptake of bacteria in vitro. For in vivo protection studies, antisera generated from mQβ‐glycan **3** conjugate significantly broadened the scope of protection, which not only improved the efficacy against *S*. Typhimurium, but also protected against *S*. Paratyphi A and *S*. Typhi.

Collectively, our findings emphasize that *O*‐acetylation is critical for broadly protective *Salmonella* glycoconjugate vaccines. The integration of chemical synthesis, optimized carrier conjugation, and cross‐species evaluation provides an effective strategy for vaccine optimization and mechanistic understanding. Our results, when confirmed in future human clinical studies, can lead to a significantly simplified broad‐spectrum vaccine design against major clinically relevant pathogenic *Salmonella enterica* serovars. In addition, as many bacterial O‐polysaccharides contain acetylation in nature, introduction of acetates in defined positions of synthetic antigens can be a promising direction to pursue for next generation vaccine development.

## Author Contributions

**Xingling Pan**: investigation, writing – original draft, writing – review and editing, data curation. **Soham Maity**: investigation. **Herbert Kavunja**: investigation. **Yuvarani Murali**: investigation. **Ting‐An Chen**: investigation. **Chang‐xin Huo**: investigation. **Scott M. Baliban**: funding acquisition, writing – review and editing. **Xuefei Huang**: conceptualization, funding acquisition, writing – review and editing, supervision, project administration.

## Conflicts of Interest

S.M. and H.K. are employees of Iaso Therapeutics. X.H. is a founder of Iaso Therapeutics.

## Supporting information




**Supporting File**: advs77776‐sup‐0001‐SuppMat.docx.

## Data Availability

The data that supports the findings of this study are available in the supplementary material of this article
