# Supplementary material for: Critical Roles of O‐Acetylation Revealed by Synthetic Trisaccharide Antigens for a Broad‐Spectrum Anti‐Salmonella Vaccine
Source: Adv Sci (Weinh). 2026 Sep 16:e77776. Online ahead of print. doi: 10.1002/advs.77776 (PMC13583234; doi:10.1002/advs.77776)
Supplement: Supplementary file 1 [file ADVS-9999-e77776-s001.docx]

**Supporting Information**

**Critical Roles of *O*-Acetylation Revealed by Synthetic Trisaccharide Antigens for a Broad-Spectrum Anti-*Salmonella* Vaccine**

Xingling Pan,^a,b^ Soham Maity,^c^ Herbert Kavunja,^c^ Yuvarani Murali,^a,b^ Ting-An Chen,^a^ Chang-xin Huo,^a,b^ Scott M. Baliban,^d^ Xuefei Huang^a,b,e*^

a.	Department of Chemistry, Michigan State University; 578 South Shaw Lane, East Lansing, MI 48824, USA.

b.	Institute for Quantitative Health Science & Engineering; Michigan State University; 578 South Shaw Lane, East Lansing, MI 48824, USA.

c.	Iaso Therapeutics Inc., 4942 Dawn Avenue, East Lansing, MI 48823, USA.

d.	Center for Vaccine Development and Global Health; University of Maryland School of Medicine; Baltimore; 685 W Baltimore St, Baltimore, MD 21201, USA.

e.	Department of Biomedical Engineering; Michigan State University; East Lansing, MI 48824, USA.

*Email: [huangxu2@msu.edu](mailto:huangxu2@msu.edu)

**Table of contents**

Animal study ethics statement	**S3**

General experimental procedures	**S3**

General synthesis procedures	**S3**

**Scheme S1**. Synthesis of monosaccharide acceptors **9** and **10**	**S4**

Synthetic procedures and characterization data	**S4**

Monitoring 3-*O*-Ac migration of **2** in PBS-D_2_O	**S8**

**Scheme S2**. Conjugation of glycans **1**-**3** with mQβ and BSA	**S11**

Synthesis of mQβ-glycan conjugates	**S11**

Synthesis of BSA-glycan conjugates	**S12**

Immunization protocol	**S12**

Evaluation of antibody titers by ELISA	**S12**

Competitive ELISA analysis	**S13**

Bacterial strains and growth conditions	**S14**

Procedure for flow cytometry	**S14**

Localization of IgG binding on the bacterial surface by TEM	**S14**

Analysis of deposition of complement component C1q to *Salmonella* strains	**S15**

Procedure for macrophage opsonophagocytosis assay	**S15**

Protection of mice from lethal challenge with *Salmonella* strains	**S16**

Computational methods and results	**S17**

**Figure S1**. ESI-TOF HRMS of representative mQβ-glycan conjugates	**S18**

**Figure S2**. MALDI-TOF mass spectra of BSA-glycan conjugates	**S20**

**Figure S3**. Transmission electron microscopy images of *S*. Paratyphi A	**S22**

**Figure S4.** Geometry optimized structure of “**1**”,“**2**”,“**3**”	**S23**

**Figure S5.** Geometry optimized structure overlay of “**1**”,“**3**”	**S24**

**Figure S6**. ^1^H NMR spectra of “**2**”, “**3**” in PBS-D_2_O	**S24**

**Figure S7.** NMR spectra of *Salmonella* COPS	**S26**

**Table S1**. NMR data of compound **11**	**S27**

**Table S2**. NMR data of compound **13**	**S27**

**Table S3**. NMR data of compound **14	S28**

**Table S4**. NMR data of compound **15**	**S29**

**Table S5**. NMR data of compound **16**	**S29**

**Table S6**. NMR data of compound **1**	**S30**

**Table S7**. NMR data of compound **2**	**S31**

**Table S8**. NMR data of compound **3**	**S32**

**References	S33**

**NMR spectra	S34**

**Animal study ethics statement**

All animal care procedures and experimental protocols have been approved by the Institutional Animal Care and Use Committee (IACUC) of Michigan State University (**protocol number:** 202200444).

**General experimental procedures**

All chemical reactions were carried out under nitrogen with anhydrous solvents in flame-dried glassware, unless otherwise noted. Glycosylation reactions were performed in the presence of molecular sieves, which were flame-dried right before the reaction under high vacuum. Glycosylation solvents were dried using a solvent purification system and used directly without further drying. Chemicals used were reagent grade as supplied except where noted. Analytical thin-layer chromatography was performed using silica gel 60 F254 glass plates. Compounds were visualized by UV light (254 nm) and by staining with a yellow solution containing Ce(NH_4_)_2_(NO_3_)_6_ (0.5 g) and (NH_4_)_6_Mo_7_O_24_•4H_2_O (24.0 g) in 6% H_2_SO_4_ (500 mL). Flash column chromatography was performed on silica gel 60 (230-400 Mesh). Optical rotations were recorded on a Perkin Elemer 341 Polarimeter (λ= 589 nm, 1 dm cell). No unexpected or unusually high safety hazards were encountered during this work.

**Mass spectrometry (MS) analysis**

ESI-MS measurements were performed according to the published procedures^[1]^ on a Water Xevo G2-XS UPLC/MS/MS and Thermo Q-Exactive UPLC/MS/MS. MALDI mass spectra was recorded on a Bruker ultrafleXtreme MALDI- TOF/TOF. The matrix used was 2,5-dihydroxy-benzoic acid (DHB).

**Characterization of anomeric stereochemistry**

The stereochemistry of the newly formed glycosidic linkages in the oligosaccharides and intermediates is determined by ^3^*J* _(H1, H2)_ through ^1^H-NMR and/or ^1^*J* _(C1, H1)_ through gHSQC 2-D NMR (without ^1^H decoupling). For galactosyl building blocks, the smaller coupling constants of ^3^*J* _(H1, H2)_ (around 3 Hz) indicate α linkages and larger coupling constants ^3^*J* _(H1, H2)_ (7.5 Hz or larger) indicate β linkages. For all glycosyl linkages, the stereochemistry can be further confirmed as larger ^1^*J* _(C1, H1)_ (around 170 Hz) suggests α linkages and smaller ^1^*J* _(C1, H1)_ (around 160 Hz) for β linkages.^[2]^

**Scheme S1**. Syntheses of the non-reducing end monosaccharide acceptors **9** and **10**

***N*-(Benzyl)benzyloxycarbonyl-3-aminopropyl 2-*O*-benzyl-3-*O*-(4-methoxybenzyl)-4,6-*O*-di-tert-butylsilylidene-α-D-galactopyranoside (8):** A mixture of donor **6**^[3]^ (500 mg, 0.79 mmol), acceptor **7** (282 mg, 0.94 mmol), and freshly activated 4 Å molecular sieves (MS) in DCM (7 mL) were stirred for 15 minutes at room temperature. The suspension was cooled to -78 °C and then NIS (267 mg, 1.19 mmol) and TfOH (8 µL, 79 µmol) were added. The reaction mixture was gradually warmed to -30 °C and stirred for 1 h at the same temperature. Then, the mixture was quenched with triethylamine, diluted with DCM and filtered. The filtrate was concentrated *in vacuo*. The obtained residue was purified by silica gel column chromatography (5:1, hexanes/ethyl acetate) to afford **8** (595 mg, 93%) as colorless syrup; ${[\alpha]}_{D}^{20}$ +101.1 (*c* 1.1, CHCl_3_); ^1^H NMR (500 MHz, CD_3_CN) δ 7.46-7.18 (m, 17H), 6.89-6.81 (m, 2H), 5.13 (s, 2H), 4.83 (d, *J* = 3.5 Hz, 1H), 4.66 (d, *J* = 11.7 Hz, 1H), 4.63-4.53 (m, 3H), 4.48 (d, *J* = 2.6 Hz, 2H), 4.07 (s, 1H), 3.97-3.81 (m, 3H), 3.77 (s, 3H), 3.75-3.70 (m, 2H), 3.69-3.62 (m, 1H), 3.41-3.30 (m, 3H), 1.84-1.73 (m, 2H), 0.99 (s, 9H), 0.99 (s, 9H). ^13^C NMR (125 MHz, CD_3_CN) δ 160.43, 140.31, 139.66, 138.49, 132.12, 130.41, 129.58, 129.53, 129.33, 128.93, 128.84, 128.73, 128.52, 128.23, 114.83, 98.36, 78.28, 77.13, 73.46, 72.18, 71.74, 67.91, 67.82, 66.36, 63.70, 56.06, 56.04, 51.65, 45.66, 29.33, 28.09, 28.07, 21.29, 21.23. HRMS (ESI): *m*/*z* calcd for C_47_H_65_N_2_O_9_Si [M+NH_4_]^+^: 829.4459, found: 829.4475.

***N*-(Benzyl)****benzyloxycarbonyl-3-aminopropyl 2-*O*-benzyl-4,6-*O*-di-tert-butylsilylidene-*α*-D-galactopyranoside (9):** To a solution of **8** (803 mg, 0.99 mmol) in DCM/H_2_O (10 mL, 10:1) was added DDQ (421 mg, 1.98 mmol), and the solution was stirred for 2 h at room temperature, then diluted with DCM. The solution was washed with saturated aqueous NaHCO_3_ and brine, dried over Na_2_SO_4_, and filtered. The filtrate was concentrated *in vacuo* and the residue was purified by silica gel column chromatography (hexanes/ethyl acetate, 5:1) to afford **9** (622 mg, 91%) as a colorless oil. ${[\alpha]}_{D}^{20}$ +30.7 (*c* 0.3, CHCl_3_); ^1^H NMR (500 MHz, CDCl_3_) δ 7.60-6.93 (m, 15H), 5.21-5.13 (m, 2H), 4.83-4.75 (m, 1H), 4.73-4.59 (m, 2H), 4.57-4.54 (m, 1H), 4.48-4.29 (m, 2H), 4.19-4.17 (m, 1H), 4.11-3.99 (m, 1H), 3.97-3.82 (m, 1H), 3.71-3.58 (m, 2H), 3.51 (s, 1H), 3.47-3.19 (m, 3H), 2.49 (d, *J* = 8.9 Hz, 1H), 1.93-1.69 (m, 2H), 1.04 (s, 9H), 0.95 (s, 9H); ^13^C NMR (125 MHz, CDCl_3_) δ 138.37, 137.97, 136.81, 128.70, 128.58, 128.56, 128.36, 128.11, 127.98, 127.38, 97.80, 77.36, 75.86, 73.72, 73.09, 69.90, 67.33, 67.07, 65.85, 50.96, 50.61, 44.71, 43.80, 28.51, 27.99, 27.69, 27.33, 23.48, 20.76. HRMS (ESI): *m*/*z* calcd for C_39_H_57_N_2_O_8_Si [M+NH_4_]^+^: 709.3884, found: 709.3891.

***N*-(Benzyl)benzyloxycarbonyl-3-aminopropyl 2,4,6-tri-*O*-benzyl-*α*-D-galactopyranoside (10):** To a solution of **8** (405 mg, 0.49 mmol) in THF (5 mL), tetrabutylammonium fluoride (TBAF) (0.5 mL, 1.0 M in THF) was added at 0 °C and stirred for 3 h. Upon reaction completion, the reaction mixture was concentrated *in vacuo* and the residue was purified by silica gel column chromatography (hexanes/ethyl acetate, 3:1). To a solution of the product in DMF (3 mL) was added BnBr (0.18 mL, 1.96 mmol) and NaH (92 mg, 1.96 mmol, 50% oil coated) and the reaction mixture was stirred at room temperature for 2 h. The reaction was quenched with saturated aqueous NH_4_Cl and then the mixture was diluted with DCM. The organic layer was washed with water and brine, dried over Na_2_SO_4_, filtered and concentrated *in vacuo.* The dry mass was dissolved in DCM/H_2_O (5 mL, 10:1) and DDQ (200 mg, 0.98 mmol) was added. The solution was stirred for 2 h at room temperature, then diluted with DCM. The solution was washed with saturated aqueous NaHCO_3_ and brine, dried over Na_2_SO_4_, and filtered. The filtrate was concentrated *in vacuo* and the residue was purified by silica gel column chromatography (hexanes/ethyl acetate, 5:1) to afford **10** (270 mg, 74% over three steps) as a colorless oil. ${[\alpha]}_{D}^{20}$ +80.7 (*c* 0.8, CHCl_3_); ^1^H NMR (500 MHz, CDCl_3_) δ 7.74-6.87 (m, 25H), 5.17 (d, *J* = 9.5 Hz, 2H), 4.87-4.72 (m, 2H), 4.68-4.44 (m, 6H), 4.43-4.37 (m, 1H), 4.07-3.96 (m, 1H), 3.93 (d, *J* = 7.3 Hz, 1H), 3.87 (d, *J* = 8.8 Hz, 1H), 3.78 (dd, *J* = 9.8, 4.6 Hz, 1H), 3.69-3.47 (m, 3H), 3.42-3.19 (m, 3H), 2.24 (d, *J* = 5.0 Hz, 1H), 1.85-1.78 (m, 2H). ^13^C NMR (125 MHz, CDCl_3_) δ 138.63, 138.31, 138.02, 136.85, 128.68, 128.65, 128.61, 128.58, 128.52, 128.51, 128.29, 128.17, 128.07, 127.96, 127.85, 127.36, 97.02, 77.49, 77.41, 77.16, 76.91, 76.67, 75.27, 73.57, 72.86, 70.24, 69.36, 69.05, 67.40, 65.84, 50.88, 50.61, 44.77, 43.87, 28.52, 28.03. HRMS (ESI): *m*/*z* calcd for C_45_H_53_N_2_O_8_ [M+NH_4_]^+^: 749.3802, found: 749.3815.

***p*-Tolyl 2-*O*-benzoyl-3,4,6-tri-*O*-benzyl-α-D-mannopyranosyl-(1→4)-2,3-*O*-isopropylidene-α-L-rhamnopyranoside (11):** A solution of donor **4**^[4]^ (66 mg, 0.1 mmol) and freshly activated 4 Å MS (200 mg) in DCM (2 mL) was stirred for 5 min at room temperature, and then cooled to -78 °C. A solution of AgOTf (77 mg, 0.3 mmol) in anhydrous Et_2_O/DCM (0.8 mL/0.2 mL) was added to reaction solution. After 5 min, orange colored *p*-TolSCl (15 μL, 97 μmol) was added to the reaction mixture through a microsyringe. The characteristic orange color of *p*-TolSCl in the reaction solution disappeared rapidly in a few seconds indicating depletion of *p*-TolSCl. After the donor was completely activated according to TLC analysis (about 5 min), a solution of acceptor **5** (28 mg, 90 μmol) with TTBP (22 mg, 90 μmol) in DCM (1.2 mL) was slowly added via a syringe. The reaction was warmed up to -20 °C under stirring in 2 h. Upon reaction completion, the reaction mixture was quenched by Et_3_N and filtered over Celite. The Celite was washed with DCM. After removal of the solvent, the desired oligosaccharide was purified by silica gel flash chromatography to afford **11** (61 mg, 90%) as colorless syrup; ${[\alpha]}_{D}^{20}$ -36.7 (*c* 1.0, CHCl_3_); ^1^H NMR (500 MHz, CDCl_3_) δ 8.12-8.08 (m, 2H), 7.59-7.54 (m, 1H), 7.45-7.21 (m, 19H), 7.18-7.13 (m, 2H), 5.69 (s, 1H), 5.61 (dd, *J* = 3.1, 1.9 Hz, 1H), 5.15 (d, *J* = 2.1 Hz, 1H), 4.94 (d, *J* = 10.6 Hz, 1H), 4.85-4.78 (m, 2H), 4.65 (d, *J* = 11.4 Hz, 1H), 4.59 (d, *J* = 10.6 Hz, 1H), 4.54 (d, *J* = 11.9 Hz, 1H), 4.32 (d, *J* = 5.5 Hz, 1H), 4.29 (d, *J* = 9.9 Hz, 1H), 4.22 (dt, *J* = 9.9, 2.2 Hz, 1H), 4.20-4.12 (m, 3H), 4.02 (dd, *J* = 10.7, 2.6 Hz, 1H), 3.78 (dd, *J* = 10.7, 1.9 Hz, 1H), 3.53 (dd, *J* = 10.0, 7.5 Hz, 1H), 2.36 (s, 3H), 1.53 (s, 3H), 1.32 (s, 3H), 1.26 (d, *J* = 6.3 Hz, 3H). ^13^C NMR (125 MHz, CDCl_3_) δ 165.99, 138.63, 138.21, 138.05, 133.29, 132.56, 130.11, 130.00, 129.89, 129.60, 128.51, 128.45, 128.44, 128.20, 128.01, 127.77, 127.73, 127.69, 127.59, 109.43, 98.45, 84.20, 80.95, 78.27, 76.64, 76.59, 75.55, 74.33, 73.61, 71.82, 71.73, 69.16, 68.74, 66.44, 28.33, 26.61, 21.27, 17.53. HRMS (ESI): *m*/*z* calcd for C_49_H_58_NO_10_S [M+NH_4_]^+^: 852.3781, found: 852.3790.

***p*-Tolyl 2,3,4,6-*O*-tetra-*O*-benzyl-α-D-mannopyranosyl-(1→4)-2-*O*-tert-butyldimethylsilyl-α-L-rhamnopyranoside (12):** Compound **11** (832 mg, 1.1 mmol) was dissolved in MeOH (10 mL), the solution was cooled to 0 °C and NaOMe (112 mg, 1.6 mmol) was added. After stirring 1 h at room temperature, the mixture was neutralized with DOWEX-H^+^ ion exchange resins, filtered and concentrated. To a solution of the debenzoylated product in DMF (5 mL) was added BnBr (0.2 mL, 1.7 mmol) and NaH (106 mg, 2.2 mmol, 50% oil coated) and the reaction mixture was stirred at room temperature for 2 h. The reaction was quenched with saturated aqueous NH_4_Cl and then the mixture was diluted with DCM. The organic layer was washed with water and brine, dried over Na_2_SO_4_, filtered and concentrated *in vacuo.* A solution of the dry residue in 80% AcOH (5 mL) was stirred at 80 °C for 2 h, and then concentrated. To a solution of the resulting mass dissolved in DCM (3 mL), imidazole (0.15 g, 2.2 mmol) and TBSCl (0.18 g, 1.2 mmol) were added. The reaction was stirred at room temperature for 2 h, then quenched with MeOH. The mixture was extracted with DCM and washed with water and brine. The organic phase was dried over Na_2_SO_4_, filtered, and concentrated under reduced pressure. The residue was purified by flash column chromatography (hexanes/ethyl acetate, 10:1), yielding compound **12** (558 mg, 56% yield over four steps). ${[\alpha]}_{D}^{20}$ +113.2 (*c* 1.5, CHCl_3_); ^1^H NMR (500 MHz, CDCl_3_) δ 7.44-7.26 (m, 20H), 7.22-7.19 (m, 2H), 7.15-7.12 (m, 2H), 5.23 (s, 1H), 4.91-4.83 (m, 2H), 4.80 (d, *J* = 12.2 Hz, 1H), 4.75-4.68 (m, 2H), 4.66 (d, *J* = 10.4 Hz, 2H), 4.55 (d, *J* = 12.2 Hz, 1H), 4.51 (d, *J* = 11.0 Hz, 1H), 4.23-4.15 (m, 2H), 4.08-4.00 (m, 1H), 3.94-3.92 (m, 2H), 3.76-3.67 (m, 4H), 3.41-3.34 (m, 2H), 2.36 (s, 3H), 1.10 (d, *J* = 6.2 Hz, 3H), 0.91 (s, 9H), 0.11 (s, 3H), 0.07 (s, 3H). ^13^C NMR (125 MHz, CDCl_3_) δ 138.51, 138.49, 138.45, 138.24, 138.09, 137.53, 132.06, 130.96, 129.92, 128.53, 128.43, 128.22, 127.96, 127.94, 127.88, 127.82, 127.70, 127.59, 100.24, 89.33, 84.33, 79.56, 75.20, 74.93, 73.69, 73.44, 73.02, 72.75, 72.36, 70.68, 69.39, 68.12, 25.94, 21.22, 18.29, 17.72, -4.41, -4.76. HRMS (ESI): *m*/*z* calcd for C_53_H_70_NO_9_SSi [M+NH_4_]^+^: 924.4541, found: 924.4563.

***p*-Tolyl 2,3,4,6-*O*-tetra-benzyl-α-D-mannopyranosyl-(1→4)-3-*O*-acetyl-2-*O*-tert-butyldimethylsilyl-α-L-rhamnopyranoside (13):** Compound **12** (500 mg, 0.6 mmol) was dissolved in pyridine (1 mL). Ac_2_O (0.1 mL) and DMAP (15 mg, 0.12 mmol) were added at 0 °C and stirred at room temperature for 3 h. The residue was purified by flash column chromatography (hexanes/ethyl acetate, 15:1), yielding compound **13** (473 mg, 90%). ${[\alpha]}_{D}^{20}$ +50.4 (*c* 0.3, CHCl_3_); ^1^H NMR (500 MHz, CDCl_3_) δ 7.50-7.25 (m, 21H), 7.22-7.20 (m, 2H), 7.16-7.14 (m, 2H), 5.17 (d, *J* = 1.7 Hz, 1H), 5.05 (dd, *J* = 9.7, 2.9 Hz, 1H), 4.98 (d, *J* = 2.1 Hz, 1H), 4.93 (d, *J* = 11.0 Hz, 1H), 4.84 (d, *J* = 12.6 Hz, 1H), 4.76 (d, *J* = 12.1 Hz, 1H), 4.73 (d, *J* = 9.3 Hz, 1H), 4.71 (d, *J* = 8.4 Hz, 1H), 4.66 (d, *J* = 11.7 Hz, 1H), 4.58 (d, *J* = 11.0 Hz, 1H), 4.52 (d, *J* = 12.1 Hz, 1H), 4.29 (dd, *J* = 2.9, 1.7 Hz, 1H), 4.20 (t, *J* = 9.7 Hz, 1H), 4.17-4.10 (m, 1H), 3.94-3.89 (m, 1H), 3.86 (d, *J* = 3.2 Hz, 1H), 3.85-3.84 (m, 1H), 3.81 (t, *J* = 9.5 Hz, 1H), 3.75-3.73 (m, 1H), 3.69 (dd, *J* = 10.7, 2.0 Hz, 1H), 2.36 (s, 3H), 2.06 (s, 3H), 1.10 (d, *J* = 6.2 Hz, 3H), 0.93 (s, 9H), 0.04 (s, 3H), 0.02 (s, 3H). ^13^C NMR (125 MHz, CDCl_3_) δ 170.39, 138.90, 138.55, 138.45, 138.30, 137.73, 132.29, 130.42, 129.93, 128.46, 128.45, 128.34, 128.33, 128.29, 128.27, 128.11, 127.91, 127.89, 127.89, 127.85, 127.81, 127.68, 127.53, 127.43, 99.10, 89.04, 79.78, 76.56, 74.95, 74.55, 74.49, 73.56, 73.09, 72.78, 72.56, 72.50, 71.62, 69.16, 68.81, 25.71, 21.46, 21.19, 18.22, -4.68, -5.03. HRMS (ESI): *m*/*z* calcd for C_55_H_72_NO_10_SSi [M+NH_4_]^+^: 966.4646, found: 966.4682.

***N*-(Benzyl)benzyloxycarbonyl-3-aminopropyl 2,3,4,6-tetra-*O*-benzyl-α-D-mannopyranosyl-(1→4)-3-*O*-acetyl-2-*O*-tert-butyldimethylsilyl-α-L-rhamnopyranosyl-(1→3)-2-*O*-benzyl-4,6-*****O*-di-tert-butylsilylidene-α-D-galactopyranoside** **(14):** A mixture of disaccharide donor **13** (378 mg, 0.4 mmol), acceptor **9** (256 mg, 0.37 mmol), and freshly activated 4 Å MS in DCM (7 mL) were stirred for 15 min at room temperature. The suspension was cooled to -78 °C and then NIS (149 mg, 0.66 mmol) and TfOH (4 µL, 44 µmol) were added. The reaction mixture was gradually warmed to -30 °C and stirred for 1 h at the same temperature. Then, the mixture was quenched with Et_3_N, diluted with DCM and filtered. The filtrate was concentrated *in vacuo*. The obtained residue was purified by silica gel column chromatography (hexanes/ethyl acetate, 3:1) to afford **14** (516 mg, 92%) as colorless syrup; ${[\alpha]}_{D}^{20}$ +31.4 (*c* 1.0, CHCl_3_); ^1^H NMR (500 MHz, CDCl_3_) δ 7.79-7.05 (m, 35H), 5.23-5.16 (m, 2H), 5.12 (dd, *J* = 9.7, 2.8 Hz, 1H), 4.97 (s, 1H), 4.91 (d, *J* = 11.1 Hz, 1H), 4.79-4.77 (m, 5H), 4.62-4.50 (m, 8H), 4.38-4.31 (m, 1H), 4.22-4.10 (m, 3H), 4.09-3.96 (m, 2H), 3.91-3.75 (m, 6H), 3.70 (d, *J* = 2.6 Hz, 1H), 3.67 (d, *J* = 10.6 Hz, 1H), 3.63-3.36 (m, 3H), 3.35-3.22 (m, 1H), 2.01 (d, *J* = 1.9 Hz, 3H), 1.94-1.68 (m, 3H), 1.08 (d, *J* = 6.2 Hz, 3H), 0.94 (s, 9H), 0.92 (s, 9H), 0.90 (s, 9H), -0.01 (s, 3H), -0.05 (s, 3H). ^13^C NMR (125 MHz, CDCl_3_) δ 170.40, 139.07, 138.59, 138.51, 138.32, 137.91, 136.78, 128.68, 128.57, 128.52, 128.49, 128.35, 128.34, 128.22, 127.98, 127.93, 127.82, 127.68, 127.54, 127.52, 127.38, 127.34, 102.68, 98.90, 98.03, 79.95, 78.84, 76.77, 74.84, 74.56, 74.09, 73.87, 73.61, 73.56, 73.24, 73.09, 72.57, 72.42, 72.34, 70.30, 68.84, 68.24, 67.31, 67.08, 65.76, 50.92, 50.50, 44.56, 43.64, 28.40, 27.93, 27.47, 27.45, 25.76, 23.47, 21.60, 20.76, 18.87, 18.13, -4.73, -4.96. HRMS (ESI): *m*/*z* calcd for C_87_H_117_N_2_O_18_Si_2_ [M+NH_4_]^+^: 1533.7840, found: 1533.7851.

**3-Aminopropyl α-D-mannopyranosyl-(1→4)-α-L-rhamnopyranosyl-(1→3)-α-D-galactopyranoside (1):** Compound **14** (100 mg, 66 µmol) was dissolved in THF (1 mL), and TBAF (1.0 M in THF, 0.1 mL) was added at 0 °C. The mixture was stirred at room temperature for 3 h. The solvent was evaporated under reduced pressure. The residue was extracted and directly dissolved in *t*-BuOH/H₂O/AcOH (5 mL, v/v/v = 4:1:0.04). Next, 20% Pd(OH)₂/C (100 mg) was added, and the reaction mixture was stirred under a hydrogen atmosphere at 30 °C for 24 h. The reaction mixture was filtered and the filtrate was concentrated *in vacuo*. The obtained residue was purified by Sephadex G-10 column (H_2_O) to afford **1** (30 mg, 82% yield over two steps) as a white solid. ${[\alpha]}_{D}^{20}$ +10.8 (*c* 0.8, H_2_O); ^1^H NMR (500 MHz, D_2_O) δ 4.86 (d, *J* = 2.1 Hz, 1H), 4.83 (s, 1H), 4.82 (s, 1H), 3.91 (dd, *J* = 3.4, 2.1 Hz, 1H), 3.89 (d, *J* = 3.7 Hz, 1H), 3.86 (dd, *J* = 3.3, 1.8 Hz, 1H), 3.83-3.77 (m, 3H), 3.77-3.71 (m, 4H), 3.68 (dd, *J* = 11.9, 3.3 Hz, 1H), 3.66-3.52 (m, 5H), 3.48-3.44 (m, 1H), 3.41 (t, *J* = 9.5 Hz, 1H), 3.07-2.93 (m, 2H), 1.89-1.83 (m, 2H), 1.16 (d, *J* = 6.4 Hz, 3H). ^13^C NMR (125 MHz, D_2_O) δ 101.90, 101.28, 98.33, 81.13, 77.34, 72.97, 70.98, 70.27, 70.24, 70.14, 68.96, 68.80, 67.98, 67.24, 66.36, 65.58, 61.04, 60.52, 37.58, 26.44, 16.77. HRMS (ESI): *m*/*z* calcd for C_21_H_40_NO_15_ [M+H]^+^: 546.2398, found: 546.2400.

**3-Aminopropyl α-D-mannopyranosyl-(1→4)-3-*O*-acetyl-α-L-rhamnopyranosyl-(1→3)-α-D-galactopyranoside (2):** To a solution of **14** (120 mg, 79 µmol) in pyridine (1 mL) was added HF•pyridine (70% HF in pyridine, 0.1 mL), and the reaction mixture was stirred for 1 h at room temperature. The reaction mixture was diluted with DCM, washed with saturated aqueous NaHCO_3_, dried over Na_2_SO_4_, and filtered. The filtrate was concentrated *in vacuo* and the residue was purified by flash column chromatography (hexanes/ethyl acetate, 2:1). To the obtained product in *t*-BuOH/H_2_O/AcOH (5 mL, v/v/v = 4:1:0.04) was added 20% Pd(OH)_2_/C (120 mg). The reaction mixture was stirred under a hydrogen atmosphere at 30 °C. The mixture was stirred for 24 h. The reaction mixture was filtered and the filtrate was concentrated *in vacuo*. The obtained residue was purified by Sephadex G-10 column (H_2_O) to afford **2** (38 mg, 85% yield over two steps) as a white solid. ${[\alpha]}_{D}^{20}$ +8.8 (*c* 0.1, H_2_O); ^1^H NMR (500 MHz, D_2_O) δ 4.98 (dd, *J* = 9.5, 3.3 Hz, 1H), 4.86 (d, *J* = 2.1 Hz, 1H), 4.83 (d, *J* = 1.8 Hz, 1H), 4.81 (d, *J* = 3.9 Hz, 1H), 4.05-4.00 (m, 1H), 3.89 (d, *J* = 3.4 Hz, 1H), 3.87-3.62 (m, 9H), 3.62-3.53 (m, 4H), 3.50 (dt, *J* = 6.1, 2.9 Hz, 1H), 3.47-3.40 (m, 1H), 3.08-2.90 (m, 2H), 2.01 (s, 3H), 1.89-1.76 (m, 2H), 1.17 (d, *J* = 6.4 Hz, 3H). ^13^C NMR (125 MHz, D_2_O) δ 173.37, 101.42, 100.90, 98.32, 77.47, 76.79, 73.04, 72.57, 70.96, 70.04, 70.00, 68.94, 68.29, 68.10, 67.15, 65.92, 65.60, 61.03, 60.33, 37.59, 26.42, 20.71, 17.04. HRMS (ESI): *m*/*z* calcd for C_23_H_42_NO_16_ [M+H]^+^: 588.2504, found: 588.2514.

**Monitoring 3-*O*-Ac migration of 2 in PBS-D_2_O**: Compound **2** was dissolved in 0.01 M PBS prepared in D_2_O (hereafter referred to as PBS-D_2_O) and incubated at room temperature. ^1^H NMR spectra were recorded after 30 min, 12 h, 24 h, and 4 days to monitor structural changes. Because the NMR spectra contained a mixture of species (Compounds **2** and **S1**) with overlapping resonances (**Figure S6**), only selected well-resolved, non-overlapping resonances were used for integration to determine the relative ratio of the species. MS analysis of the sample showed only the target MS with no MS signals corresponding to cleavage of *O*-acetyl observed. HRMS (ESI): *m*/*z* calcd for C_23_H_42_NO_16_ [M+H]^+^: 588.2504, found: 588.2510.

Compound **2** in PBS-D_2_O for 30 min: The selected diagnostic resonances of the Ac-transferred species (compound **S1**) showed a relative integral of approximately 0.37 when the corresponding resonances of compound **2** were normalized to 1.00. ^1^H NMR (600 MHz, PBS-D_2_O) δ 5.12 (dd, *J* = 3.4, 2.1 Hz), 5.00 (s, 1H), 4.06 (dd, *J* = 9.5, 3.5 Hz), 2.10 (s) were assigned to compound **S1**, whereas the resonances at 5.08 (dd, *J* = 9.6, 3.3 Hz), 4.97 (d, *J* = 2.0 Hz), 4.15-4.10 (m), 2.11 (s) corresponded to compound **2**.

Compound **2** in PBS-D_2_O for 12 h: The selected diagnostic resonances of compound **S1** showed a relative integral of approximately 0.65 when the corresponding resonances of compound **2** were normalized to 1.00. ^1^H NMR (600 MHz, PBS-D_2_O) δ 5.12 (dd, *J* = 3.6, 1.8 Hz), 5.00 (d, *J* = 1.5 Hz), 4.06 (dd, *J* = 9.6, 3.6 Hz), 2.10 (s) were assigned to compound **S1**, whereas the resonances at 5.08 (dd, *J* = 9.6, 3.3 Hz), 4.99-4.96 (m), 4.15-4.11 (m), 2.11 (s) corresponded to compound **2**.

Compound **2** in PBS-D_2_O for 24 h: The selected diagnostic resonances of compound **S1** showed a relative integral of approximately 0.74 when the corresponding resonances of compound **2** were normalized to 1.00. ^1^H NMR (600 MHz, PBS-D_2_O) δ 5.12 (dd, *J* = 3.6, 1.9 Hz), 5.00 (d, *J* = 1.9 Hz), 4.06 (dd, *J* = 9.6, 3.5 Hz), 2.10 (s) were assigned to compound **S1** , whereas the resonances at 5.08 (dd, *J* = 9.6, 3.3 Hz), 4.97 (d, *J* = 2.2 Hz), 4.15-4.11 (m), 2.11 (s) corresponded to compound **2**.

Compound **2** in PBS-D_2_O for 4 days: The selected diagnostic resonances of compound **S1** showed a relative integral of approximately 0.72 when the corresponding resonances of compound **2** were normalized to 1.00. ^1^H NMR (600 MHz, PBS-D_2_O) δ 5.12 (dd, *J* = 3.5, 1.8 Hz), 5.00 (d, *J* = 1.9 Hz), 4.06 (dd, *J* = 9.5, 3.5 Hz), 2.10 (s) were assigned to compound **S1**, whereas the resonances at 5.08 (dd, *J* = 9.6, 3.3 Hz), 4.97 (d, *J* = 2.1 Hz), 4.15-4.10 (m), 2.11 (s) corresponded to compound **2**.

***p*-Tolyl 2,3,4,6-*O*-tetra-*O*-benzyl-α-D-mannopyranosyl-(1→4)-2,3-di-*O*-acetyl-α-L-rhamnopyranoside (15):** Compound **11** (832 mg, 1.1 mmol) was dissolved in MeOH (10 mL), the solution was cooled to 0 °C and NaOMe (112 mg, 1.6 mmol) was added. After stirring 1 h at room temperature, the mixture was neutralized with DOWEX-H^+^ ion exchange resins, filtered and concentrated. To a solution of the debenzoylated product in DMF (5 mL) was added BnBr (0.2 mL, 1.7 mmol) and NaH (103 mg, 2.2 mmol, 50% oil coated) and the reaction mixture was stirred at room temperature for 2 h. The reaction was quenched with MeOH and the solvents were evaporated to dryness. A solution of the dry mass dissolved in 80% AcOH (5 mL) was stirred at 80 °C for 2 h, and then concentrated. To a solution of the resulting mass in pyridine (5 mL), Ac_2_O (0.1 mL) was added. The reaction was stirred at room temperature for 2 h, then quenched with MeOH. The mixture was concentrated under reduced pressure. The residue was purified by flash column chromatography (hexanes/ethyl acetate, 8:1), yielding compound **15** (713 mg, 74% yield over four steps); ${[\alpha]}_{D}^{20}$ +91.4 (*c* 0.9, CHCl_3_); ^1^H NMR (500 MHz, CDCl_3_) δ 7.65-7.23 (m, 20H), 7.20-7.16 (m, 2H), 7.15-7.11 (m, 2H), 5.47 (dd, *J* = 3.4, 1.7 Hz, 1H), 5.27 (s, 1H), 5.14 (dt, *J* = 9.8, 2.4 Hz, 1H), 4.93 (d, *J* = 1.8 Hz, 1H), 4.90 (d, *J* = 11.0 Hz, 1H), 4.83 (d, *J* = 12.6 Hz, 1H), 4.73-4.63 (m, 4H), 4.57-4.51 (m, 2H), 4.22-4.13 (m, 1H), 4.07 (t, *J* = 9.7 Hz, 1H), 3.90-3.86 (m, 1H), 3.83 (dd, *J* = 9.4, 3.1 Hz, 1H), 3.80 (dd, *J* = 10.7, 4.2 Hz, 1H), 3.74 (t, *J* = 9.5 Hz, 1H), 3.72-3.68 (m, 2H), 2.34 (s, 3H), 2.05 (s, 3H), 1.99 (s, 3H), 1.08 (d, *J* = 6.4 Hz, 3H). ^13^C NMR (125 MHz, CDCl_3_) δ 170.19, 170.04, 138.69, 138.51, 138.42, 138.32, 138.15, 132.49, 130.00, 129.70, 128.51, 128.40, 128.34, 128.10, 127.91, 127.87, 127.80, 127.77, 127.61, 127.56, 99.38, 86.02, 79.71, 76.91, 75.05, 74.71, 74.67, 73.55, 72.91, 72.73, 72.69, 71.56, 70.50, 69.27, 69.06, 21.24, 21.08, 20.93, 17.98. HRMS (ESI): *m*/*z* calcd for C_56_H_60_NO_11_S [M+NH_4_]^+^: 894.3887, found: 894.3909.

***N*-(Benzyl)benzyloxycarbonyl-3-aminopropyl 2,3,4,6-tetra-*O*-benzyl-α-D-mannopyranosyl-(1→4)-2,3-di-*O*-acetyl-α-L-rhamnopyranosyl-(1→3)-2,4,6-tri-*O*-benzyl-α-D-galactopyranoside (16):** A mixture of disaccharide donor **15** (437 mg, 0.50 mmol), acceptor **7** (311 mg, 0.45 mmol), and freshly activated 4 Å MS in DCM (8 mL) were stirred for 15 min at room temperature. The suspension was cooled to -78 °C and then NIS (169 mg, 0.75 mmol) and TfOH (4.4 µL, 50 µmol) were added. The reaction mixture was gradually warmed to -30 °C and stirred for 1 h at the same temperature. Then, the mixture was quenched with Et_3_N, diluted with DCM and filtered. The filtrate was concentrated *in vacuo*. The obtained residue was purified by silica gel column chromatography (hexanes/ethyl acetate, 5:1) to afford **16** (621 mg, 92%) as colorless syrup; ${[\alpha]}_{D}^{20}$ +87.3 (*c* 1.0, CHCl_3_); ^1^H NMR (500 MHz, CDCl_3_) δ 7.43-7.21 (m, 42H), 7.21-7.13 (m, 3H), 5.46 (dd, *J* = 3.4, 1.8 Hz, 1H), 5.24 (dd, *J* = 9.8, 3.4 Hz, 1H), 5.21 (s, 1H), 5.20-5.17 (m, 2H), 4.99 (d, *J* = 11.1 Hz, 1H), 4.94 (d, *J* = 2.2 Hz, 1H), 4.88 (d, *J* = 11.1 Hz, 1H), 4.83 (d, *J* = 12.6 Hz, 1H), 4.75-4.64 (m, 5H), 4.62-4.57 (m, 2H), 4.57-4.44 (m, 7H), 4.13 (t, *J* = 7.6 Hz, 1H), 4.08 (t, *J* = 9.7 Hz, 1H), 4.04-3.99 (m, 1H), 3.94-3.92 (m, 1H), 3.87-3.82 (m, 2H), 3.82-3.74 (m, 3H), 3.73-3.65 (m, 3H), 3.64-3.50 (m, 3H), 3.42-3.25 (m, 3H), 2.02 (s, 3H), 2.00 (s, 3H), 1.91-1.75 (m, 2H), 1.10 (d, *J* = 6.1 Hz, 3H). ^13^C NMR (125 MHz, CDCl_3_) δ 170.13, 169.83, 156.73, 156.23, 138.71, 138.49, 138.41, 138.32, 138.19, 137.92, 136.80, 128.62, 128.55, 128.53, 128.51, 128.48, 128.44, 128.41, 128.38, 128.29, 128.12, 128.07, 128.01, 127.94, 127.91, 127.85, 127.83, 127.80, 127.78, 127.75, 127.70, 127.59, 127.51, 127.42, 99.35, 98.79, 97.18, 79.66, 77.57, 77.10, 76.82, 75.35, 75.00, 74.69, 74.63, 73.70, 73.51, 73.04, 72.83, 72.64, 70.17, 70.03, 69.55, 69.12, 68.96, 68.48, 67.24, 65.79, 50.91, 50.60, 44.66, 43.70, 28.45, 27.95, 21.12, 20.84, 18.26. HRMS (ESI): *m*/*z* calcd for C_89_H_101_N_2_O_19_ [M+NH_4_]^+^: 1501.6999, found: 1501.6731.

**3-Aminopropyl α-D-mannopyranosyl-(1→4)-2,3-di-*O*-acetyl-α-L-rhamnopyranosyl-(1→3)-α-D-galactopyranoside (3):** To a solution of **16** (130 mg, 88 µmol) in *t*-BuOH/H_2_O/AcOH (5 mL, v/v/v = 4:1:0.04) was added 20% Pd(OH)_2_/C (130 mg), and the reaction mixture was stirred under a hydrogen atmosphere at 30 °C. The mixture was stirred for 24 h. The reaction mixture was filtered and the filtrate was concentrated *in vacuo*. The obtained residue was purified by Sephadex G-10 column (H_2_O) to afford **3** (51 mg, 92%) as a white solid. ${[\alpha]}_{D}^{20}$ +6.8 (*c* 0.11, H_2_O); ^1^H NMR (500 MHz, D_2_O) δ 5.20 (dd, *J* = 3.3, 1.9 Hz, 1H), 5.05 (dd, *J* = 9.7, 3.3 Hz, 1H), 4.91 (d, *J* = 1.9 Hz, 1H), 4.81 (d, *J* = 1.9 Hz, 1H), 4.80 (d, *J* = 4.0 Hz, 1H), 3.94-3.86 (m, 2H), 3.82 (dd, *J* = 10.2, 4.0 Hz, 1H), 3.78 (d, *J* = 2.2 Hz, 1H), 3.77-3.69 (m, 4H), 3.65-3.58 (m, 2H), 3.58-3.53 (m, 4H), 3.53-3.49 (m, 1H), 3.45-3.39 (m, 1H), 3.03-2.89 (m, 2H), 1.98 (s, 3H), 1.90 (s, 3H), 1.85-1.78 (m, 2H), 1.18 (d, *J* = 6.3 Hz, 3H). ^13^C NMR (125 MHz, D_2_O) δ 173.26, 172.83, 100.90, 98.85, 98.32, 77.77, 76.49, 73.03, 70.93, 70.71, 70.06, 69.99, 69.70, 68.84, 68.38, 67.04, 65.91, 65.71, 61.03, 60.32, 37.65, 26.38, 20.50, 20.03, 16.90. HRMS (ESI): *m*/*z* calcd for C_25_H_44_NO_17_ [M+H]^+^: 630.2609, found: 630.2609.

When compound **3** was incubated in PBS-D_2_O as performed for compound **2** for up to 4 days, no changes were observed in the NMR (**Figure S6**) or MS spectra, suggesting that no detectable acetyl migration or cleavage occurred under the experimental condition.

**Scheme S2**. Conjugation of glycans **1**-**3** with mQβ and BSA.

**Synthesis of mQβ-glycan conjugates**

A solution of adipate bisNHS ester **17** (16 mg in 0.5 mL DMF, 0.045 mmol) was added into a solution of trisaccharide **1** (5 mg in 0.5 mL DMF, 0.009 mmol), which was followed by addition of di-isopropylethylamine (DIPEA, 2 μL, 0.009 mmol). The reaction mixture was stirred at room temperature for 2 h. After completion of the reaction, the solvent was removed under reduced pressure. The resulting residue was suspended in DCM and centrifuged. Under these conditions, product **1ꞌ** precipitated, whereas excess bis-NHS ester **17** remained soluble in DCM. The supernatant was carefully decanted, and the solid was washed with DCM three times in the same manner to remove residual **17**. The resulting solid was dried to give pure compound **1ꞌ**. To a solution of 5 mg/mL mQβ particle (Qβ A38K/A40C/D102C) in PBS (100 mM, pH = 7.4), **1ꞌ** (20 equiv to mQβ subunits) was added. The solution was gently mixed overnight at room temperature on a tube rotator. The protein was recovered with a 0.5 mL Amicon filter (MWCO = 100 kDa) under 13,000 g and washed with PBS (100 mM, pH = 7.4) for 4 times. Conjugation reactions of mQβ with **2ꞌ and 3ꞌ** was conducted in similar manners. The final conjugates were analyzed by HRMS (**Figure S1**).

**Synthesis of BSA-glycan conjugates**

To a solution of 10 mg/mL BSA in PBS (100 mM, pH = 7.4) **1ꞌ** (60 equiv to per BSA molecule) was added. The solution was gently mixed and nutated at room temperature overnight. The protein was recovered with a 0.5 mL Amicon filter (MWCO = 30 kDa) under 13,000 g and washed with Milli-Q water for 4 times, then lyophilize it and store in -80 °C. Conjugation reactions of BSA with **2ꞌ** and **3ꞌ** were conducted in similar manners. The final conjugates were analyzed by MALDI (**Figure. S2**).

**Procedure for mouse immunization**

Pathogen-free C57BL/6 female mice aged 6 weeks were purchased from Charles River and maintained in the University Laboratory Animal Resources facility of Michigan State University. All animal experiments were performed in accordance with the guidelines of the Institutional Animal Care and Use Committee (IACUC) of Michigan State University (**protocol number:** 202200444). C57BL/6 mice were immunized subcutaneously under the scruff with 0.2 mL mQβ-glycan construct (15 µg glycan per dose) formulated with 100 µg Alum (CAS # 21645-51-2, Alhydrogel® adjuvant 2%, InvivoGen) in PBS (without Mg^2+^ and Ca^2+^). Mice in group I-III were immunized on days 0,14 and 42. Mice in group IV received an escalating immunization schedule on days 0, 2, 4, 6, 8, 10, 12, 14 and 42. The glycan doses (µg per injection) were 0.06, 0.129, 0.348, 0.945, 2.568, 5.136, 8.964, 11.5, and 15, respectively, with corresponding Alum amounts (µg) of 0.4, 0.62, 2.32, 6.3, 17.12, 34.24, 59.76, 79, and 100.Vaccine constructs were adsorbed onto adjuvant by incubating the antigen with Alum at room temperature for 30 min prior to immunization. Blood samples were collected on days 0, 7, 21 and 49.

**Procedure for rabbit immunization.**

Rabbit immunization studies were performed by ProSci Inc (Poway, CA). Two New Zealand rabbits were injected subcutaneously on day 0 with 0.1 mL mQβ-glycan (glycan **1**, glycan **2** or glycan **3**) constructs (at 15 μg glycan) as emulsions in Complete Freund’s Adjuvant according to manufacturer’s instructions. Immunization procedures were performed on day 0, 14, 28 and 42. Blood samples were collected on days 0, and 35. The terminal bleeding was performed on day 49.

**Evaluation of antibody titers by ELISA**

Immulon 4 HBX 384 well plates (Thermo Fisher 8755) were coated with a solution of BSA-glycan conjugates or COPS (10 μg/mL, 50 μL/well) in PBS buffer. For whole-cell bacterial coating, bacteria were grown to log phase and adjusted to an OD_600_ of 0.2 in PBS. A total of 50 μL per well was added to the plates and incubated at 4 °C overnight. The plate was washed with PBST (4 × 100 μL), blocked with 1% BSA/PBS (100 μL/well) for 1 h at room temperature. The liquid was discarded, and the plates were washed with PBST (4 × 100 μL), and incubated with serial dilutions of antisera from immunized mice or rabbits in 0.1% BSA/PBS (50 μL/well, 3 wells for each dilution). The plates were incubated for 2 h and then liquid was discarded. The plates were washed with PBST (4 × 100 μL). A 1:2000 dilution of HRP-conjugated goat anti-mouse IgG (Cat # 115-035-003), 1:5000 dilution of goat anti-rabbit IgG (Cat # 111-035-003) (all from Jackson Immuno Research Laboratory) in 0.1% BSA/PBS (50 μL) was added and incubated for 1 h. The liquid was discarded, and the plates were washed with PBST (4 × 100 μL). A solution of enzymatic substrate was prepared by dissolving 3,3′,5,5′-tetramethylbenzidine (TMB) (10 mg) in a mixture of DMSO (4 mL) and citric acid buffer (36 mL) in a 50 mL centrifuge tube covered with aluminum foil. H_2_O_2_ (40 μL) was added and the mixture was homogenized by vortexing. Then, 75 μL of substrate solution was added to each well, and the reaction was incubated for 15 minutes. To stop the reaction, 25 μL of 0.5 M H_2_SO_4_ was added. OD_450_ was immediately recorded and fitted into 4PL nonlinear logistic model via GraphPad Prism 10 with least squares algorism. The endpoint titer was determined through regression analysis, where the log10 dilution was plotted against optical density. It was reported as the highest dilution fold (ELISA units) that yielded an optical absorbance at the background threshold level.

**Competitive ELISA analysis.**

Immulon 4 HBX 384 well plates (Thermo Fisher 8755) were coated with a solution of BSA-glycan **1** conjugates (10 μg/mL, 50 μL/well) in PBS buffer. A total of 50 μL per well was added to the plates and incubated at 4 °C overnight. The plate was washed with PBST (4 × 100 μL), blocked with 1% BSA/PBS (100 μL/well) for 1 h at room temperature. The liquid was discarded, and the plates were washed with PBST (4 × 100 μL), and incubated with serial dilutions of antisera from immunized mice or rabbits in 0.1% BSA/PBS (50 μL/well, 3 wells for each dilution). Serial dilutions of free glycans **1**-**3** were added to the plate. Rabbit sera collected at day 49 posy-immunization with mQβ-glycan **1** were diluted in PBS containing 1% BSA then add to the plate. The total volume is 50 μL, the sera final dilution is 1:1,000,000, and final glycan concentration ranging from 0.004-68μg/ml. The plates were incubated for 2 h and then liquid was discarded. The plates were washed with PBST (4 × 100 μL). A 1:5000 dilution of HRP-conjugated goat anti-rabbit IgG (Cat # 111-035-003, Jackson Immuno Research Laboratory) in 0.1% BSA/PBS (50 μL) was added and incubated for 1 h. The liquid was discarded, and the plates were washed with PBST (4 × 100 μL). A solution of enzymatic substrate was prepared by dissolving TMB (10 mg) in a mixture of DMSO (4 mL) and citric acid buffer (36 mL) in a 50 mL centrifuge tube covered with aluminum foil. H_2_O_2_ (40 μL) was added and the mixture was homogenized by vortexing. Then, 75 μL of substrate solution was added to each well, and the reaction was incubated for 15 minutes. To stop the reaction, 25 μL of 0.5 M H_2_SO_4_ was added. OD_450_ was immediately recorded and fitted into 4PL nonlinear logistic model via GraphPad Prism 10 with least squares algorism. Normalize % absorbance = [(OD_sample_ - OD_backgroud_)/(OD _maximum binding_-OD_backgroud_)] ×100. IC_50_ was defined as the glycan concentration that produced 50% inhibition of maximal antibody and coating antigen binding as calculated from normalized competition curves using log(inhibitor) vs normalized response-variable slope regression.

**Bacterial strains and growth conditions**

The bacterial strains used in this study included wild type *S*. Enteritidis R11, *S*. Typhimurium I77, *S*. Typhimurium D65, *S*. Paratyphi A ATCC 9150, *S*. Typhi Ty2, and *S*. Newport Chile 361, as well as genetically engineered reagent strains for COPS isolation: CVD 1902 (*S*. Paratyphi A), CVD 1925 pSEC10-*wzzB* (*S*. Typhimurium), CVD 1943 (*S*. Enteritidis), and CVD 1966 (*S*. Newport). All the subtypes of *Salmonella* have been described previously^[5]^ and all strains were maintained in Hi-Soy (HS) bacteriological media (5 g/L sodium chloride, 10 g/L soytone [Teknova, CA], 5 g/L Hyyest [Sigma Aldrich, MO]) at 37 °C as previously described.^[6]^

*Salmonella* COPS used as ELISA antigens were purified from fermentation cultures as described^[7]^ previously by extraction with acetic acid and purification by sequential tangential flow filtration, anion exchange chromatography, and ammonium sulfate precipitation.

**Procedure for flow cytometry**

A single colony of *S.* Paratyphi A ATCC 9150 was grown in HS broth overnight at 37 °C with shaking at 220 rpm. The following day, bacteria were adjusted to an OD_600_ of 0.4 and placed on ice. 250 µL of bacteria were washed once with flow buffer (1% heat-inactivated FBS in PBS) and incubated with different dilution of pooled rabbit sera (heat-inactivated for 30 min at 56 °C) for 1 h at 4 °C. Rabbit sera tested included post-immune (D49) or pre-immune sera (D0). Bacteria were then washed two times with flow buffer, followed by incubation with 1 µg/mL of FITC-conjugated donkey anti-rabbit IgG (Cat # 406403, Biolegend) diluted in flow buffer for 1 h at 4 °C. Bacteria were then washed twice with PBS, fixed with 2% formaldehyde, and read using BD Accuri C6 with 1 × 10^4^ events recorded. As a negative control, bacteria were incubated with secondary antibody alone. The other strains were conducted in similar manners.

**Localization of IgG binding on the bacterial surface by immunogold negative-stain TEM**

The binding of IgG antibodies to bacterial surface antigens was analyzed by negative staining and transmission electron microscopy (TEM) using immunogold labeling. A single colony of *S*. Paratyphi A ATCC 9150 was used to inoculate HS broth and grown overnight at 37 °C with shaking at 220 rpm. The following day, log-phase cultures were prepared by diluting the overnight culture 1:100 into fresh HS broth and incubating at 37 °C with shaking at 220 rpm until the OD_600_ reached 0.8. Bacteria were harvested by centrifugation, washed once with PBS, resuspended in PBS, and adjusted to an OD_600_ of 0.5. Heat-inactivated serum (56 °C for 30 min) was mixed with the bacterial suspension to obtain a final serum dilution of 1:5 and incubated at room temperature for 1 h with gentle mixing. Unbound serum was removed by centrifugation, and the bacterial pellet was washed once and resuspended in PBS. ImmunoGold Reagents EM Goat Anti-Rabbit IgG (10 nm; SKU 25108, Electron Microscopy Sciences) were added to the bacterial suspension at a final dilution of 1:20 and incubated at room temperature for 1 h. For TEM analysis, samples were adsorbed onto carbon-coated copper grids for 5 min. Grids were then washed with distilled water and negatively stained with 0.75% (wt/vol) uranyl formate (CAS # 16984-59-1, Electron Microscopy Sciences) for 30 seconds. Images were acquired using a JEOL 1400Flash transmission electron microscope.

**Analysis of deposition of complement component C1q to *Salmonella* strains**

The ability of each test serum to deposit C1q onto *Salmonella* OPS antigen was tested in triplicates in three parallel wells. Samples of each serum were heated at 56 °C for 30 min to inactivate the endogenous complement in the serum. This is important because the endogenous complement within each sample may vary. Heat-inactivation of endogenous complement removes this variable. To each individual well was added each serum at an appropriate dilution (50 μL) made in gelatin-veronal buffer (GVB, Boston BioProducts IBB-300X) with a complement source added (PelFreeze IgM Depleted Rabbit Complement Pooled Serum diluted to 2.5% in GVB; 50 μL). The plates were incubated for 1 h on a shaker at 37 °C, and washed 3 times with PBST. Subsequently, affinity‐purified goat anti‐human complement C1q antibody (Cedarlane, Inc, CL7341AP, 100 μL, 1:5000 dilution in GVB) was added to each well and incubated for 1 h under ambient temperature. This was washed three times with PBST, after which anti‐goat IgG‐alkaline phosphatase secondary antibody (100 μL, 1:5000 dilution in GVB) was added. Plates were incubated for 1 h at ambient temperature. After the plates were washed 3 times, di-sodium 4‐nitrophenyl phosphate (1mg/mL, 100 μL) in substrate buffer (1.6 g/L NaHCO_3_, 2.92 g/L Na_2_CO_3_, and 100 mg/L MgCl_2_) was added to each well and the alkaline phosphatase‐mediated color development was allowed to proceed for 30 min at 37 °C in the dark. OD_405_ for each plate was measured in a 96-well plate reader. Titers were calculated by simple linear regression as the OD_405_ values (fold of dilution on the X axis) where the curve crossed the Y axis at an OD reading determined by adding 3 times the standard deviation to the mean upper OD limits of control wells with pre-immune sera.

**Procedure for macrophage opsonophagocytosis assay**

THP-1 (human acute monocytic leukemia cell line, ATCC TIB-202) were maintained in Roswell Park Memorial Institute (RPMI) 1640 (Thermo Fisher, #11875093) with L-glutamine and 10% v/v heat-inactivated FBS (Cat# P711S500, Lonza), hereafter referred to as R10. For routine subculture, R10 was supplemented with 100 U/mL penicillin/100 μg/mL streptomycin (Cat# 15140122, Gibco) to prevent contamination. Cells were maintained at a density between 2 × 10^5^ and 1 × 10^6^ cells/mL. For differentiation, THP-1 cells were harvested, counted, and seeded into 24-well plates at 1 × 10^6^ cells/well. Phorbol 12-myristate 13-acetate (PMA; CAS# 16561-29-8, MedChemExpress) was added to a final concentration of 100 nM. Plates were gently mixed and incubated for 24 h at 37 °C in a humidified atmosphere containing 5% CO_2_. Following differentiation, partially confluent THP-1 monolayers were washed three times with R10 to remove PMA-containing medium. The media was then replaced with 500 μL of fresh complete RPMI-1640 without PMA, and cells were incubated for an additional 24-48 h. Once a stable monolayer had formed, the culture medium was replaced with R10. Bacteria were prepared in the following manner: a single colony of *S*. Enteritidis R11 was used to inoculate an overnight HS broth culture (37 °C, 220 rpm). The following day, log-phase cultures were obtained by diluting the overnight culture into fresh HS broth and incubating under the same conditions. Bacterial cultures were adjusted to an OD600 of 0.1 in PBS, corresponding to approximately 8 × 10^4^ CFU/μL. For opsonization, 22.5 μL of the diluted bacteria, 20 μL of pooled rabbit sera, and 55.5 μL of PBS were combined in microcentrifuge tubes and incubated for 20 min at room temperature with periodic gentle mixing. The sera for this experiment were collected from Qβ-glycan immunized rabbits (pre-immunization and day 49 post-immunization) and Qβ-immunized rabbits (day 49 post-immunization). Subsequently, 6 μL of the opsonized bacteria (1 × 10^5^ CFU) were added to duplicate wells of the THP-1 monolayers in 24-well plate. Plates were centrifuged at 500 × g for 2 min to facilitate bacterial contact with cells, followed by incubation at 37 °C / 5% CO_2_ for 50 min. After incubation, wells were washed once with sterile R10, and 500 μL of R10 supplemented with 100 μg/mL of gentamicin (Cat# 15750060, Gibco) was added to each well. Plates were returned to the 37 °C /5% CO_2_ incubator for 1 h. After gentamicin treatment, wells were washed three times with sterile R10, and cells were lysed with 500 μL of sterile water for 10 min with shaking at 250 rpm. Wells were then scraped with a pipette tip to ensure complete lysis. Lysates were then serially diluted in PBS and plated on HS agar plates to determine viable counts.

All other bacterial strains were prepared and tested using the same experimental protocol.

**Protection of mice from lethal challenge with *Salmonella* strains:**

Pre-immunization and day 49 post-immunization rabbit sera were pooled within each group (n = 2 rabbits per group), heat-inactivated at 56 °C for 30 min and diluted in PBS. Naïve 8-week-old female CD-1 mice (Charles River Laboratories, USA) were injected intraperitoneally (i.p.) with diluted rabbit sera or PBS as a control. Five to six hours later, mice were challenged i.p. with the indicated *Salmonella* strains. For *S*. Paratyphi A ATCC 9150 and *S*. Typhi Ty2, recipient mice were injected i.p. with 200 µL of rabbit sera diluted 1:10 in PBS or PBS alone. Mice were then challenged with 1×10^6^ CFU of *S*. Paratyphi A ATCC 9150 or 2.5 ×10^4^ CFU of *S*. Typhi Ty2, prepared from overnight HS agar plates and suspended in 100 µL of sterile 10% hog gastric mucin (Sigma-Aldrich, Cat # M1778). For *S*. Enteritidis R11, naïve CD-1 mice received pooled pre-immune or day 49 post-immunization rabbit sera diluted 1:100 in PBS and administered i.p.. Mice were challenged 5-6 h later with 1.3 ×10^6^ CFU of *S*. Enteritidis R11 prepared from overnight HS plates and diluted in PBS. For *S*. Typhimurium I77, the same experimental protocol was followed, except that pooled rabbit sera were diluted 1:200 in PBS and mice were challenged with 5 ×10^5^ CFU of *S*. Typhimurium I77. Following challenge, mice were monitored three times daily for up to 21 days. Animals that became moribund and/or exhibited sustained body weight loss of ≥ 20% were euthanized and recorded as having succumbed to infection. At 21 days post-challenge, all surviving mice were euthanized, and their kidneys and spleens were aseptically collected and homogenized. Bacterial burdens were determined by serial dilution and plating on HS agar plates to confirm clearance of the challenge bacteria.

**Computational methods and results**

All computational results were done by the Gaussian 16 program. Chemcraft 1.6 was used to visualize structures. Calculations were performed adopting the B3LYP functional,^[8]^ and the 6-311++G(d,p) basis set for structure optimizations, single point geometries calculations, and free energy calculations. Calculations were conducted on closed-shell singlet. Geometry optimizations were also performed without symmetry constraints at 298.15 K in water with unscaled vibrational frequencies. Single point calculation of the optimized geometry also included solvent (SMD-water)^[9]^ and dispersion (GD3BJ)^[10]^ correction.

a)

b)

c)

**Figure S1**. ESI-TOF HRMS spectra of the mQβ-glycan **1**, mQβ-glycan **2** and mQβ-glycan **3** conjugates. a) Mass spectrometry analysis of the mQβ-glycan **1** conjugate showed an average loading of 635 trisaccharides on each capsid. The peaks observed showed a sequential mass shift of ~655 corresponding to the addition of one glycan **1**. (b) Mass spectrometry analysis of the mQβ-glycan **2** conjugate showed an average loading of 498 trisaccharides on each capsid. The peaks observed showed a sequential mass shift of ~698 corresponding to the addition of one glycan **2**. (c) Mass spectrometry analysis of the mQβ-glycan **3** conjugate showed an average loading of 595 trisaccharides on each capsid. The peaks observed showed a sequential mass shift of ~741 corresponding to the addition of one glycan **3**. The average loading was calculated using the following formula: (sum of the intensities of peaks of mQβ conjugate multiplied by the number of glycan attached for the peak)/(sum of the intensities of peaks of mQβ conjugate and that of the unmodified mQβ).

a)

b)

c)

d)

**Figure S2**. MALDI-TOF characterization of BSA-Glycans. (a) BSA protein only; (b) The molecular weight of BSA shifted from 66,005 Da to 77,504 Da after conjugation, indicating ~18 glycan **1** units per BSA molecule; (c) The molecular weight of BSA shifted from 66,005 Da to 80,626 Da after conjugation, indicating ~20 glycan **2** units per BSA molecule; (d) The molecular weight of BSA shifted from 66,005 Da to 79,079 Da after conjugation, indicating ~17 glycan **3** units per BSA molecule. The difference of MW before and after conjugation divided by the MW of Glycans (glycan **1**: 655; glycan **2**: 698; glycan **3**:741) gave the average loading of copies of Glycan per BSA molecule.

a) b)

c) d)

**Figure S3**. **Transmission electron microscopy (TEM) visualization of antibody binding to *S.* Paratyphi A ATCC 9150.** Representative TEM images showing ImmunoGold labeling of bacteria incubated with (a) pre-immune sera (1:5 dilution), (b) mQβ-glycan **1** immune serum (1:5 dilution), (c) mQβ-glycan **2** immune serum (1:5 dilution), and (d) mQβ-glycan **3** immune serum (1:5 dilution). For each vaccine group, sera from two rabbits were pooled. A subset of particles is indicated with red circles to aid visualization. The scale bar is 500 nm.

a)

b)

c)

**Figure S4.** (a) Geometry optimized structure of glycan **1**. Two conformers were identified, with glycan **1**-1 being 1.1 kcal/mol lower in energy than glycan **1**-2. (b) Geometry optimized structure of glycan **2**. Two conformers were identified, with glycan **2**-1 being 4.8 kcal/mol lower in energy than glycan **2**-2. (c) Geometry optimized structure of glycan **3**. Four conformers were identified; glycan **3**-1 is 0.9, 3.4, and 7.9 kcal/mol lower in energy than glycan **3**-2, glycan **3**-3, and glycan **3**-4, respectively. The *O*-acetyl group is highlighted with a yellow circle.

Red atom: oxygen; white atom: hydrogen; grey atom: carbon; blue atom: nitrogen.

**Figure S5.** Glycan **1**-1 and glycan **3**-1were selected for molecular superposition. The molecular structures of glycan **1** and glycan **3** were overlapped using Chemcraft 1.6, and are shown from two perspectives, revealing a maximum interatomic deviation of approximately 3.9 Å. Hydrogen atoms are not displayed for clarity. Atom colors are as follows: red, oxygen atoms of glycan **3**; pink, oxygen atoms of glycan **1**; grey, carbon atoms of glycan **3**; purple, carbon atoms of glycan **1**; blue, nitrogen atoms of glycan **3**; orange, nitrogen atoms of glycan **1**.

a)

b)

**Figure S6.** ^1^H NMR spectra of a) glycan **2** in D_2_O and after incubation in PBS-D_2_O for 30 min, 12 h, 24 h, and 4 days; b) glycan **3** in D_2_O and after incubation in PBS-D_2_O for 30 min, 24 h, and 4 days.

**Figure S7.** ^1^H NMR spectra of COPS isolation from CVD 1902 (*S*. Paratyphi A), CVD 1925 pSEC10-wzzB (*S*. Typhimurium), CVD 1943 (*S*. Enteritidis) were recorded in D_2_O at 500 MHz and at room temperature, demonstrating differences in the relative abundance of *O*-acetyl groups on rhamnose residues.

**Table S1**. NMR data of compound **11**

| Position | ^1^H (ppm) | ^13^C (ppm) | ^1^H-^1^H COSY | HMBC (H-C) |
| --- | --- | --- | --- | --- |
| 1 | 5.69 (s, 1H) | 84.2 | H-2 | C-2, C-5 |
| 2 | 4.32 (d, *J* = 5.5 Hz, 1H) | 76.64 | H-1, H-3 | C-1, C-3 |
| 3 | 4.14 | 76.59 | H-2, H-4 | C-2, C-4 |
| 4 | 3.53 (dd, *J* = 10.0, 7.5 Hz, 1H) | 80.95 | H-3, H-5 | C-2, C-3, C-5, C-6, C-1' |
| 5 | 4.18 | 66.44 | H-4, H-6 | C-4, C-6 |
| 6 | 1.26 (d, *J* = 6.3 Hz, 3H) | 17.53 | H-5 | C-4, C-5 |
| 1' | 5.15 (d, *J* =2.1 Hz,1H) | 98.4 | H-2' | C-4, C-2', C-3', C-5' |
| 2' | 5.61 (dd, *J* = 3.1, 1.9 Hz,1H) | 69.16 | H-1', H-3' | C-3', C-4' |
| 3' | 4.16 | 78.27 | H-2', H-4' | C-2', C-4' |
| 4' | 4.29 (d, *J* = 9.9 Hz, 1H) | 74.44 | H-3', H-5' | C-3', C-5', C-6' |
| 5' | 4.22 (dt, *J* = 9.9, 2.2 Hz, 1H) | 71.82 | H-4', H-6' | C-4' |
| 6' | 4.02 (dd, *J* = 10.7, 2.6 Hz, 1H);  3.78 (dd, *J* = 10.7, 1.9 Hz, 1H) | 68.74 | H-5' | C-4', C-5' |

**Table S2**. NMR data of compound **13**

| Position | ^1^H (ppm) | ^13^C (ppm) | ^1^H-^1^H COSY | HMBC (H-C) |
| --- | --- | --- | --- | --- |
| 1 | 5.17 (d, *J* = 1.7 Hz, 1H) | 89.04 | H-2 | C-2, C-3, C-5 |
| 2 | 4.29 (dd, *J* =2.9, 1.7 Hz, 1H) | 71.62 | H-1, H-3 | C-3, C-4 |
| 3 | 5.05 (dd, *J* = 9.7, 2.9 Hz, 1H) | 73.09 | H-2, H-4 | C-4 |
| 4 | 3.81 (t, *J* = 9.5 Hz, 1H) | 76.56 | H-3, H-5 | C-2, C-3, C-5, C-6, C-1' |
| 5 | 4.17-4.10 (m, 1H) | 69.16 | H-4, H-6 | C-1, C-3, C-4, C-6 |
| 6 | 1.10 (d, *J* = 6.2 Hz, 3H) | 18.22 | H-5 | C-4, C-5 |
| 1' | 4.98 (d, *J* = 2.1 Hz, 1H) | 99.1 | H-2' | C-4, C-2', C-3', C-4', C-5' |
| 2' | 3.75-3.73 (m, 1H) | 74.55 | H-1', H-3' | C-3', C-4' |
| 3' | 3.85-3.84 (m, 1H) | 79.78 | H-2', H-4' | C-2', C-4' |
| 4' | 3.94-3.89 (m, 1H) | 72.5 | H-3', H-5' | C-5' |
| 5' | 4.20 (t, *J* = 9.7 Hz, 1H) | 74.95 | H-4', H-6' | C-3', C-4', C-6' |
| 6' | 3.86 (d, *J* = 3.2 Hz, 1H);  3.69 (dd, *J* = 10.7, 2.0 Hz, 1H) | 68.81 | H-5' | C-4', C-5' |

**Table S3**. NMR data of compound **14**

| Position | ^1^H (ppm) | ^13^C (ppm) | ^1^H-^1^H COSY | HMBC (H-C) |
| --- | --- | --- | --- | --- |
| 1 | 4.59 | 98.03 | H-2 | C-2 |
| 2 | 3.84 | 73.56 | H-1, H-3 | C-3 |
| 3 | 3.86 | 78.84 | H-2, H-4 | C-2, C-4 |
| 4 | 4.38-4.31 (m, 1H) | 74.09 | H-3, H-5 | C-3 |
| 5 | 4.17 | 72.42 | H-4, H-6 | C-4 |
| 6 | 4.14; 4.06 | 67.08 | H-5 | C-5 |
| 7 | 4.52 | 50.92, 50.50 | H-1, H-8 |  |
| 8 | 1.84 | 27.45 | H-7, H-9 |  |
| 9 | 3.42; 3.28 | 44.56, 43.64 | H-8 | C-7 |
| 1' | 4.79 | 102.68 | H-2' | C-3 |
| 2' | 4.16 | 70.3 | H-1', H-3' | C-3', C-4' |
| 3' | 5.12 (dd, *J* = 9.7, 2.8 Hz, 1H) | 73.24 | H-2', H-4' | C-4' |
| 4' | 3.78 | 76.77 | H-3', H-5' | C-3', C-5', C-6', C-1'' |
| 5' | 4.00 | 68.24 | H-4', H-6' | C-4', C-6' |
| 6' | 1.08 (d, *J* = 6.2 Hz, 3H) | 18.87 | H-5' | C-4', C-5' |
| 1'' | 4.97 (s, 1H) | 98.9 | H-2'' | C-4', C-5'', C-3'', C-2'' |
| 2'' | 3.70 (d, *J* =2.6 Hz, 1H) | 73.87 | H-1'', H-3'' | C-3'', C-4'' |
| 3'' | 3.79 | 79.95 | H-2'', H-4'' | C-4'', C-2'', C-1'' |
| 4'' | 4.19 | 74.56 | H-3'', H-5'' | C-3'', C-5'', C-6'' |
| 5'' | 3.89 | 72.34 | H-4'', H-6'' | C-4'' |
| 6'' | 3.83;  3.67 (d, *J* = 10.6 Hz, 1H) | 68.84 | H-5'' | C-4'', C-5'' |

**Table S4**. NMR data of compound **15**

| Position | 1H (ppm) | 13C(ppm) | 1H-1H COSY | HMBC(H-C) |
| --- | --- | --- | --- | --- |
| 1 | 5.27 (s, 1H)) | 86.02 | H-2 | C-2, C-3, C-5 |
| 2 | 5.14 (dt, *J* = 9.8, 2.4 Hz, 1H) | 70.5 | H-1, H-3 | C-3, C-4 |
| 3 | 5.47(dd, *J* = 3.4, 1.7 Hz, 1H) | 71.56 | H-2, H-4 | C-2, C-4 |
| 4 | 3.74 (t, *J* = 9.5 Hz, 1H) | 76.91 | H-3, H-5 | C-2, C-5, C-1' |
| 5 | 4.22-4.13 (m, 1H) | 69.06 | H-4, H-6 | C-6 |
| 6 | 1.08 (d, *J* = 6.4 Hz, 3H) | 17.98 | H-5 | C-4, C-5 |
| 1' | 4.93 (d, *J* = 1.8 Hz, 1H) | 99.38 | H-2' | C-4, C-2', C-3', C-5' |
| 2' | 3.71 | 74.71 | H-1', H-3' | C-3', C-4' |
| 3' | 3.83 (dd, *J* = 9.4, 3.1 Hz, 1H) | 79.71 | H-2', H-4' | C-2', C-4', C-5' |
| 4' | 4.07 (t, *J* = 9.7 Hz, 1H) | 74.67 | H-3', H-5' | C-2', C-3', C-5', C-6' |
| 5' | 3.90-3.86 (m, 1H) | 72.69 | H-4', H-6' | C-4' |
| 6' | 3.80 (dd, *J* = 10.7, 4.2 Hz, 1H);  3.70 | 69.27 | H-5' | C-4', C-5' |

**Table S5**. NMR data of compound **16**

| Position | ^1^H (ppm) | ^13^C (ppm) | ^1^H-^1^H COSY | HMBC (H-C) |
| --- | --- | --- | --- | --- |
| 1 | 4.70 | 97.18 | H-2 | C-2 |
| 2 | 4.04-3.99 (m, 1H) | 77.57 | H-1, H-3 |  |
| 3 | 4.13 (t, *J* = 7.6 Hz, 1H) | 75.35 | H-2, H-4 | C-2, C-1' |
| 4 | 3.84 | 77.1 | H-3, H-5 | C-2, C-3 |
| 5 | 3.94-3.92 (m, 1H) | 72.42 | H-6 | C-6 |
| 6 | 3.54; 3.61 | 68.96 | H-5 | C-5 |
| 7 | 4.51 (m, 2H) | 50.91, 50.60 | H-8 | C-8, C-9 |
| 8 | 1.91-1.75 (m, 2H) | 27.45 | H-7, H-9 | C-7, C-9 |
| 9 | 3.38 (m, 1H); 3.30 (m, 1H) | 44.66, 43.70 | H-8 | C-7, C-8 |
| 1' | 5.21 (s, 1H) | 98.79 | H-2' | C-3, C-2', C-5' |
| 2' | 5.46 (dd, *J* = 3.4, 1.8 Hz, 1H) | 70.03 | H-1', H-3' | C-1', C-3', C-4' |
| 3' | 5.24 (dd, *J* = 9.8, 3.4 Hz, 1H) | 70.17 | H-2', H-4' | C-2', C-4' |
| 4' | 3.69 | 76.82 | H-3', H-5' | C-3', C-5', C-6', C-1'' |
| 5' | 3.79 | 68.48 | H-4', H-6' | C-3', C-4', C-6' |
| 6' | 1.10 (d, *J* = 6.1 Hz, 3H) | 18.26 | H-5' | C-4', C-5' |
| 1'' | 4.94 (d, *J* = 2.2 Hz, 1H) | 99.35 | H-2'' | C-4', C-2'', C-3'', C-5'' |
| 2'' | 3.70 (m, 1H) | 74.69 | H-1'', H-3'' | C-1'', C-3'', C-4'', C-5'' |
| 3'' | 3.77 (m, 1H) | 79.66 | H-2'', H-4'' | C-2'', C-4'', C-5'' |
| 4'' | 4.08 (t, *J* = 9.7 Hz, 1H) | 74.63 | H-3'', H-5'' | C-3'', C-5'', C-6'' |
| 5'' | 3.84 (m, 1H) | 72.64 | H-4'', H-6'' | C-4'', C-6'' |
| 6'' | 3.67 (m, 1H); 3.74 (m, 1H) | 69.12 | H-5'' | C-5'' |

**Table S6**. NMR data of compound **1**

| Position | ^1^H (ppm) | ^13^C (ppm) | ^1^H-^1^H COSY | HMBC (H-C) |
| --- | --- | --- | --- | --- |
| 1 | 4.82 (s, 1H) | 98.33 | H-2 | C-5, C-8 |
| 2 | 3.81 | 72.97 | H-1, H-3 | C-3 |
| 3 | 3.71 | 77.34 | H-2, H-4 | C-1, C-2, C-1' |
| 4 | 3.89 (d, *J* = 3.7 Hz, 1H) | 68.96 | H-3, H-5 | C-2, C-3 |
| 5 | 3.74 | 67.98 | H-6 | C-1, C-4, C-6 |
| 6 | 3.58 | 61.04 | H-5 | C-4, C-5 |
| 7 | 3.74, 3.48-3.44 (m, 1H) | 65.58 | H-8 | C-1, C-8, C-9 |
| 8 | 1.89-1.83 (m, 2H) | 26.43 | H-7, H-9 | C-7, C-9 |
| 9 | 3.07-2.93 (m, 2H) | 37.58 | H-8 | C-7, C-8 |
| 1' | 4.86 (d, *J* = 2.1 Hz, 1H) | 101.90 | H-2' | C-3, C-5' |
| 2' | 3.91 (dd, *J* = 3.4, 2.1 Hz, 1H) | 70.24 | H-1', H-3' | C-3', C-4' |
| 3' | 3.78 | 70.98 | H-2', H-4' | C-4' |
| 4' | 3.41 (t, *J* = 9.5 Hz, 1H) | 81.14 | H-3', H-5' | C-3', C-1'' |
| 5' | 3.75 | 68.81 | H-4', H-6' | C-4', C-6' |
| 6' | 1.16 (d, *J* = 6.4 Hz, 3H) | 16.77 | H-5' | C-4', C-5' |
| 1'' | 4.83 (s, 1H) | 101.28 | H-2'' | C-4', C-2'', C-5'' |
| 2'' | 3.86 (dd, *J* = 3.3, 1.8 Hz, 1H) | 70.27 | H-1'', H-3'' | C-3'', C-4'' |
| 3'' | 3.68 (dd, *J* = 11.9, 3.3 Hz, 1H) | 70.14 | H-2'', H-4'' | C-4'' |
| 4'' | 3.55 | 66.36 | H-3'', H-5'' | C-3'', C-5'', C-6'' |
| 5'' | 3.78 | 67.24 | H-4'', H-6'' | C-4'', C-6'' |
| 6'' | 3.61, 3.72 | 60.52 | H-5'' | C-5'' |

**Table S7**. NMR data of compound **2**

| Position | ^1^H (ppm) | ^13^C (ppm) | ^1^H-^1^H COSY | HMBC (H-C) |
| --- | --- | --- | --- | --- |
| 1 | 4.81 (d, *J* = 3.9 Hz, 1H) | 98.32 | H-2 | C-3, C-5, C-8 |
| 2 | 3.82 | 67.15 | H-1, H-3 | C-3 |
| 3 | 3.72 | 77.47 | H-2, H-4 | C-1, C-2, C-1' |
| 4 | 3.89 (d, *J* = 3.4 Hz, 1H) | 68.94 | H-3, H-5 | C-2, C-3 |
| 5 | 3.77 | 70.96 | H-6 | C-1, C-4, C-6 |
| 6 | 3.56 | 61.03 | H-5 | C-4, C-5 |
| 7 | 3.74 , 3.47-3.40 (m, 1H) | 65.6 | H-8 | C-1, C-8, C-9 |
| 8 | 1.89-1.76 (m, 2H) | 26.42 | H-7, H-9 | C-7, C-9 |
| 9 | 3.08-2.90 (m, 2H) | 37.59 | H-8 | C-7, C-8 |
| 1' | 4.86 (d, *J* = 2.1 Hz, 1H) | 101.42 | H-2' | C-3, C-3', C-5' |
| 2' | 4.05-4.00 (m, 1H) | 68.1 | H-1', H-3' | C-3', C-4' |
| 3' | 4.98 (dd, *J* = 9.5, 3.3 Hz, 1H) | 72.57 | H-2', H-4' | C-4' |
| 4' | 3.68 | 76.79 | H-3', H-5' | C-3', C-1'' |
| 5' | 3.84 | 68.29 | H-4', H-6' | C-4', C-6' |
| 6' | 1.17 (d, *J* = 6.4 Hz, 3H) | 17.04 | H-5' | C-4', C-5' |
| 1'' | 4.83 (d, *J* = 1.8 Hz, 1H) | 100.9 | H-2'' | C-4', C-2'', C-5'' |
| 2'' | 3.79 | 70.0 | H-1'', H-3'' | C-3'', C-4'' |
| 3'' | 3.50 | 73.04 | H-2'', H-4'' | C-4'' |
| 4'' | 3.55 | 70.04 | H-3'', H-5'' | C-3'', C-5'', C-6'' |
| 5'' | 3.57 | 65.92 | H-4'', H-6'' | C-4'', C-6'' |
| 6'' | 3.64 | 60.33 | H-5'' | C-4'', C-5'' |

**Table S8**. NMR data of compound **3**

| Position | ^1^H (ppm) | ^13^C(ppm) | ^1^H-1H COSY | HMBC(H-C) |
| --- | --- | --- | --- | --- |
| 1 | 4.80 (d, *J* = 4.0 Hz, 1H) | 98.32 | H-2 | C-3, C-5, C-8 |
| 2 | 3.82 (dd, *J* = 10.2, 4.0 Hz, 1H) | 67.04 | H-1, H-3 | C-3 |
| 3 | 3.74 | 77.77 | H-2, H-4 | C-1, C-2, C-4, C-1' |
| 4 | 3.89 | 68.84 | H-3, H-5 | C-2, C-3 |
| 5 | 3.76 | 70.93 | H-6 | C-1, C-4, C-6 |
| 6 | 3.54 | 61.03 | H-5 | C-4, C-5 |
| 7 | 3.70 (m, 1H), 3.45-3.39 (m, 1H) | 65.71 | H-8 | C-1, C-8, C-9 |
| 8 | 1.85-1.78 (m, 2H) | 26.38 | H-7, H-9 | C-7, C-9 |
| 9 | 3.03-2.89 (m, 2H) | 37.65 | H-8 | C-7, C-8 |
| 1' | 4.91 (d, *J* = 1.9 Hz, 1H) | 98.85 | H-2' | C-3, C-2', C-3', C-5' |
| 2' | 5.20 (dd, *J* = 3.3, 1.9 Hz, 1H) | 69.7 | H-1', H-3' | C-3', C-4' |
| 3' | 5.05 (dd, *J* = 9.7, 3.3 Hz, 1H) | 70.71 | H-2', H-4' | C-2', C-4' |
| 4' | 3.72 | 76.49 | H-3', H-5' | C-3', C-5', C-1'' |
| 5' | 3.90 | 68.38 | H-4', H-6' | C-3', C-4', C-6' |
| 6' | 1.18 (d, *J* = 6.3 Hz, 3H) | 16.9 | H-5' | C-4', C-5' |
| 1'' | 4.81 (d, *J* = 1.9 Hz, 1H) | 100.9 | H-2'' | C-4', C-2'', C-3'', C-5'' |
| 2'' | 3.78 (d, *J* = 2.2 Hz, 1H) | 69.99 | H-1'', H-3'' | C-1'', C-4'', C-5'' |
| 3'' | 3.53-3.49 (m, 1H) | 73.03 | H-2'', H-4'' | C-4'', C-5'' |
| 4'' | 3.55 | 70.06 | H-3'', H-5'' | C-3'', C-5'' |
| 5'' | 3.56 | 65.91 | H-4'', H-6'' | C-4'', C-6'' |
| 6'' | 3.61 | 60.32 | H-5'' | C-5'' |

**References**

[1]	C. E. Doneanu, W. Chen, J. C. Gebler, *Anal. Chem.* **2009**, *81*, 3485-3499.

[2]	K. Bock, C. Pedersen, *J. Chem. Soc., Perkin Trans. 2* **1974**, 293-297.

[3]	X. Pan, C. Huo, S. Maity, H. Kavunja, R. Moszyk, C. Talbot, S. M. Baliban, X. Huang, *Angew. Chem. Int. Ed. Engl.* **2026**, *65*, e7740689.

[4]	Y. H. Lin, B. Ghosh, K. K. Mong, *Chem Commun (Camb)* **2012**, *48*, 10910-10912.

[5]	aS. M. Tennant, J. Y. Wang, J. E. Galen, R. Simon, M. F. Pasetti, O. Gat, M. M. Levine, *Infect. Immun.* **2011**, *79*, 4175-4185; bS. Nasrin, F. J. Fuche, K. T. Sears, J. A. Jones, M. M. Levine, R. Simon, S. M. Tennant, *Vaccines* **2021**, *9*; cD. Dhara, S. M. Baliban, C. X. Huo, Z. Rashidijahanabad, K. T. Sears, S. T. Nick, A. K. Misra, S. M. Tennant, X. Huang, *Chem. Eur. J.* **2020**, *26*, 15953-15968; dS. M. Baliban, S. Shridhar, K. Luo, J. Kolasny, S. Hyun, Z. Zhao, S. M. Tennant, A. S. Cross, *J. Infect. Dis.* **2025**, *232*, 133-142.

[6]	T. C. Darton, S. M. Baliban, B. Curtis, D. Toema, S. M. Tennant, M. M. Levine, M. F. Pasetti, R. Simon, *PLoS Negl. Trop. Dis.* **2018**, *12*, e0006522.

[7]	S. M. Baliban, M. Yang, G. Ramachandran, B. Curtis, S. Shridhar, R. S. Laufer, J. Y. Wang, J. Van Druff, E. E. Higginson, N. Hegerle, K. M. Varney, J. E. Galen, S. M. Tennant, A. Lees, A. D. MacKerell, Jr., M. M. Levine, R. Simon, *PLoS Negl Trop Dis* **2017**, *11*, e0005493.

[8]	aA. D. Becke, *Phys. Rev. A* **1988**, *38*, 3098-3100; bJ. P. Perdew, *Phys. Rev. B* **1986**, *33*, 8822-8824.

[9]	A. V. Marenich, C. J. Cramer, D. G. Truhlar, *J. Phys. Chem. B* **2009**, *113*, 6378-6396.

[10]	S. Grimme, S. Ehrlich, L. Goerigk, *J. Comput. Chem.* **2011**, *32*, 1456-1465.

NMR spectra
